# Genome-wide methylation sequencing of paired primary and metastatic cell lines identifies common DNA methylation changes and a role for EBF3 as a candidate epigenetic driver of melanoma metastasis

**DOI:** 10.18632/oncotarget.14042

**Published:** 2016-12-20

**Authors:** Aniruddha Chatterjee, Peter A Stockwell, Antonio Ahn, Euan J Rodger, Anna L Leichter, Michael R Eccles

**Affiliations:** ^1^ Department of Pathology, Dunedin School of Medicine, University of Otago, Dunedin, New Zealand; ^2^ Maurice Wilkins Centre for Molecular Biodiscovery, Auckland, New Zealand; ^3^ Department of Biochemistry, University of Otago, Dunedin, New Zealand

**Keywords:** melanoma, metastasis, DNA methylation, bisulfite sequencing, cancer epigenome

## Abstract

Epigenetic alterations are increasingly implicated in metastasis, whereas very few genetic mutations have been identified as authentic drivers of cancer metastasis. Yet, to date, few studies have identified metastasis-related epigenetic drivers, in part because a framework for identifying driver epigenetic changes in metastasis has not been established. Using reduced representation bisulfite sequencing (RRBS), we mapped genome-wide DNA methylation patterns in three cutaneous primary and metastatic melanoma cell line pairs to identify metastasis-related epigenetic drivers. Globally, metastatic melanoma cell lines were hypomethylated compared to the matched primary melanoma cell lines. Using whole genome RRBS we identified 75 shared (10 hyper- and 65 hypomethylated) differentially methylated fragments (DMFs), which were associated with 68 genes showing significant methylation differences. One gene, *Early B Cell Factor 3* (*EBF3*), exhibited promoter hypermethylation in metastatic cell lines, and was validated with bisulfite sequencing and in two publicly available independent melanoma cohorts (*n* = 40 and 458 melanomas, respectively). We found that hypermethylation of the *EBF3* promoter was associated with increased *EBF3* mRNA levels in metastatic melanomas and subsequent inhibition of DNA methylation reduced *EBF3* expression. RNAi-mediated knockdown of *EBF3* mRNA levels decreased proliferation, migration and invasion in primary and metastatic melanoma cell lines. Overall, we have identified numerous epigenetic changes characterising metastatic melanoma cell lines, including *EBF3*-induced aggressive phenotypic behaviour with elevated *EBF3* expression in metastatic melanoma, suggesting that *EBF3* promoter hypermethylation may be a candidate epigenetic driver of metastasis.

## INTRODUCTION

Cutaneous melanoma is a highly aggressive malignancy that originates from melanocytes and accounts for 75% of skin cancer related deaths [1]. Although globally cancer related deaths have decreased over the last two decades, the death rate from melanoma continues to increase [2]. Melanoma patients diagnosed at an early stage with full resection of primary melanoma have a high 5-year survival rate, but > 90% of melanoma patient- related deaths are due to metastasis, and their 5-year survival rate is poor [3, 4]. Therefore, better understanding of melanoma metastasis is important to develop treatments to inhibit metastasis.

Remarkable progress has been made over the last two decades in understanding the genetic basis of melanoma tumorigenesis. Genetic mutations (including driver mutations, such as in *BRAF* and *NRAS*) and other accompanying non-pathogenic mutations (passenger mutations) have been identified [5]. However, very few mutations have been identified that play a key role in contributing to metastasis [6]. Melanoma is strongly predisposed to metastasis, but relatively little is known about the role that epigenetic changes play during the metastatic processes, during which the tumor cells acquire new properties [7].

DNA methylation is a stable epigenetic modification, first shown to be altered in cancer more than three decades ago [8]. Deregulation of the tumor epigenome, such as promoter-specific hypermethylation and global hypomethylation, has been observed in almost every cancer type [9, 10], and is now considered to be a major molecular contributor to tumorigenesis.

The detection of aberrant promoter methylation patterns or global alterations in methylation patterns in primary tumors compared to healthy individuals has been a major focus of cancer epigenetics studies in the last decade [9], including studies in melanoma, which have focused on identifying DNA methylation changes associated with the initial events of tumor formation [11, 12]. While some epigenetic changes have been shown to accompany metastasis of melanoma [3, 11–15], most studies have used relatively low-resolution technologies such as 450K methylation arrays, where a small number of CpG sites (biased towards gene promoters) were investigated. As a result, large numbers of CpG sites have not been analyzed, and many genomic regions were not profiled in these platforms, particularly gene body methylation, which has recently been implicated as a key epigenetic regulator of gene expression during carcinogenesis [16].

Extensive molecular heterogeneity exhibited by tumor cells may complicate epigenetic profiling. For instance, clonal evolution of tumor cells and the presence of stromal cells both lead to intra-tumoral heterogeneity, while heterogeneity is also observed between multiple different sites of tumor metastasis in a single patient (inter-tumoral), as well as between tumors from different patients (inter-patient). As DNA methylation profiles are highly tissue-specific, and can vary extensively from cell to cell in a tissue, this heterogeneity contributes to the complexity in identifying epigenetic factors involved in tumor progression, and in identifying novel epigenetic prognostic or therapeutic biomarkers [6]. In order to minimise heterogeneity, tumor cell lines may be used in the first instance in an initial approach to decrease complexity when identifying driver epigenetic alterations, because cell lines are relatively less heterogeneous than tissues, and therefore have fewer epigenotypes than tumor tissues overall.

Although several mutational studies have been performed in matched primary and metastatic cancers and cell lines, very few genome-scale DNA methylation sequencing studies of similarly matched samples have been carried out. More importantly, methylation changes identified in the context of metastasis have frequently lacked functional studies, an important link between epigenetic changes and phenotype. In the present study we sought to identify epigenetic changes associated with metastasis, so as to identify putative “epigenetic drivers” of metastasis. To this end, we used sequencing based genome-wide reduced representation bisulfite sequencing (RRBS) DNA methylation analysis of metastatic melanoma cell lines, together with paired primary melanoma cell lines derived from the same patients, to identify metastasis-associated DNA methylation changes, which were then followed up by functional analysis of observed changes at the mRNA level.

## RESULTS

### Generating RRBS methylomes of paired melanoma cell lines

To identify epigenetic changes occurring in metastatic melanoma, compared to the primary tumor of origin, we profiled whole genome methylation in three primary cutaneous melanoma and three matched metastatic melanoma cell lines. The primary melanoma cell lines were WM115, Hs688(A).T, and WM75, and the matching metastatic melanoma cell lines were WM266-4, Hs688(B).T, and WM373, respectively. In addition, Mel-ST was included as a normal melanocyte cell line (Supplementary Table S1). To assess reproducibility, we included replicate libraries for Mel-ST and WM115 cell lines. A total of 172.5 million sequenced reads (length = 100 bp) were produced from 9 RRBS libraries and mapped to the reference human genome (GRCh37) using Bismark [17]. The unique alignment efficiency of sequenced reads ranged from 54% to 67.7% (median = 65.5%, Supplementary Table S2).

### Genome-scale analysis of the melanoma methylome

**Figure 1:**
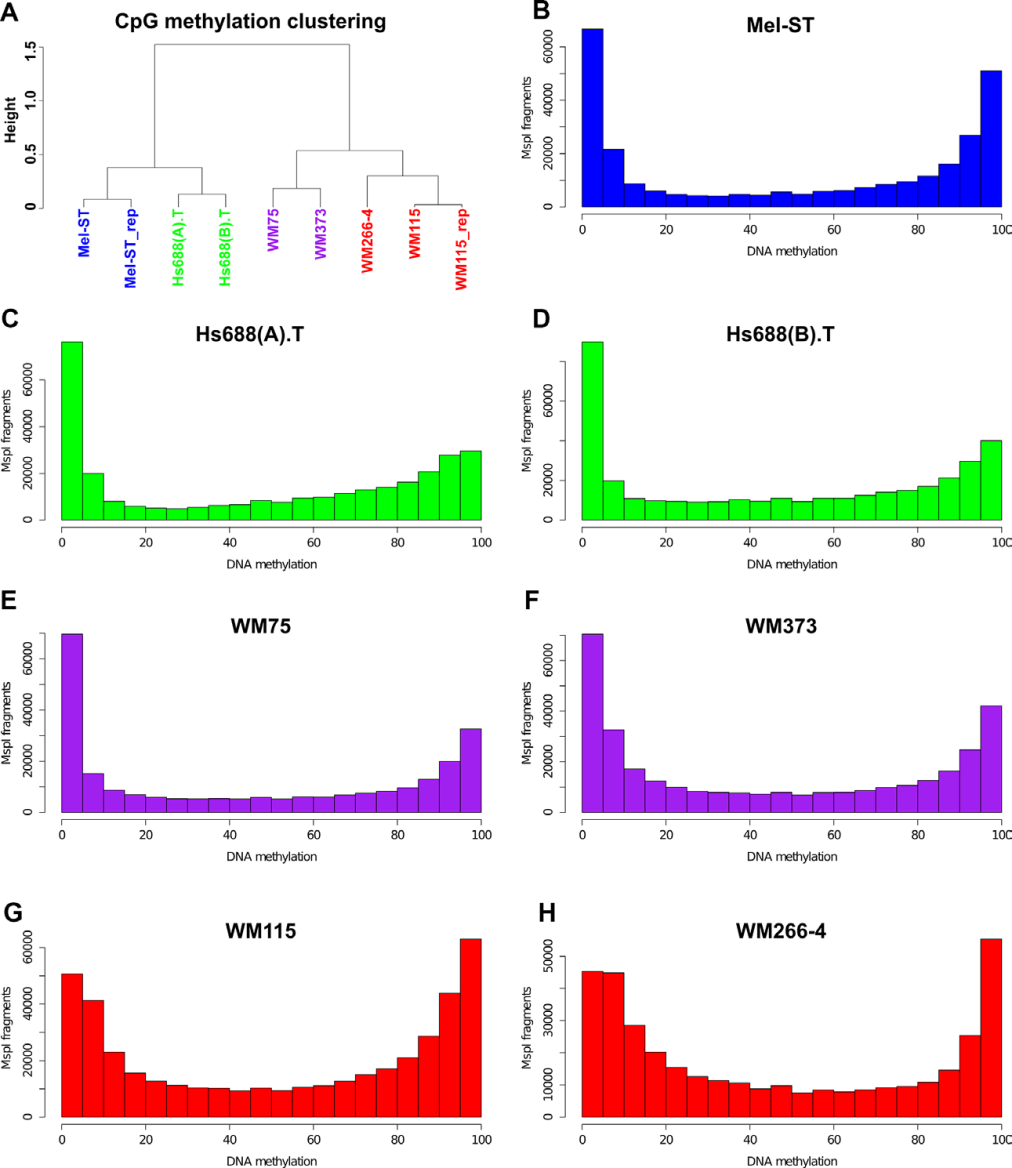
Global methylation patterns and clustering of melanoma cell lines (**A**) Hierarchical clustering of the commonly covered CpG sites (with 10-fold or higher coverage) between the cell lines. (**B**–**H**) distribution of CpG methylation patterns for 7 melanoma cell lines. For each cell line the MspI fragments that had ≥ 10 reads for ≥ 2 CpG sites in the fragment were filtered and plotted as a histogram using R studio.

High levels of technical reproducibility were observed in the Mel-ST1 and Mel-ST2 (Pearson's *r* = 0.96) and WM-115 and WM-115-2 (*r* = 0.98) replicate libraries (Supplementary Figure S1, only CpG sites covered by ≥ 10 reads were analysed). Therefore data from the replicates were combined for further analysis. The global mean methylation in these cell lines ranged from 45.13% to 53.26% (median = 47.29) (Supplementary Table S3). We observed a bimodal pattern of methylation (i.e., either hypo or hypermethylation) in the cell lines, similar to the methylation patterns described for normal somatic cells [18]. WM266-4 and WM115 cells showed a notable level of intermediate methylation (Figure 1B–1H). The non-CpG methylation in these cell lines was very low (median = 3.3%, as indicated by Bismark). Hierarchical clustering of the methylation profiles (CpG sites covered by ≥ 10 reads) revealed that primary cell lines closely resembled their corresponding metastatic matching cell lines. However, each cell line pair was distinct and clustered separately from the others (Figure 1A). Analysis of the DNA methylation distribution between different genomic elements (gene body, promoters and inter-genic) indicated that there were some differences, particularly between WM115 and WM266-4 (Supplementary Table S4), but unlike a previous study, which used an array-based technique [14], we did not observe metastasis-specific loss of gene body methylation in melanoma cell lines (Supplementary Figure S2 and Supplementary Table S4).

### Differential methylation landscapes in melanoma metastasis

**Figure 2:**
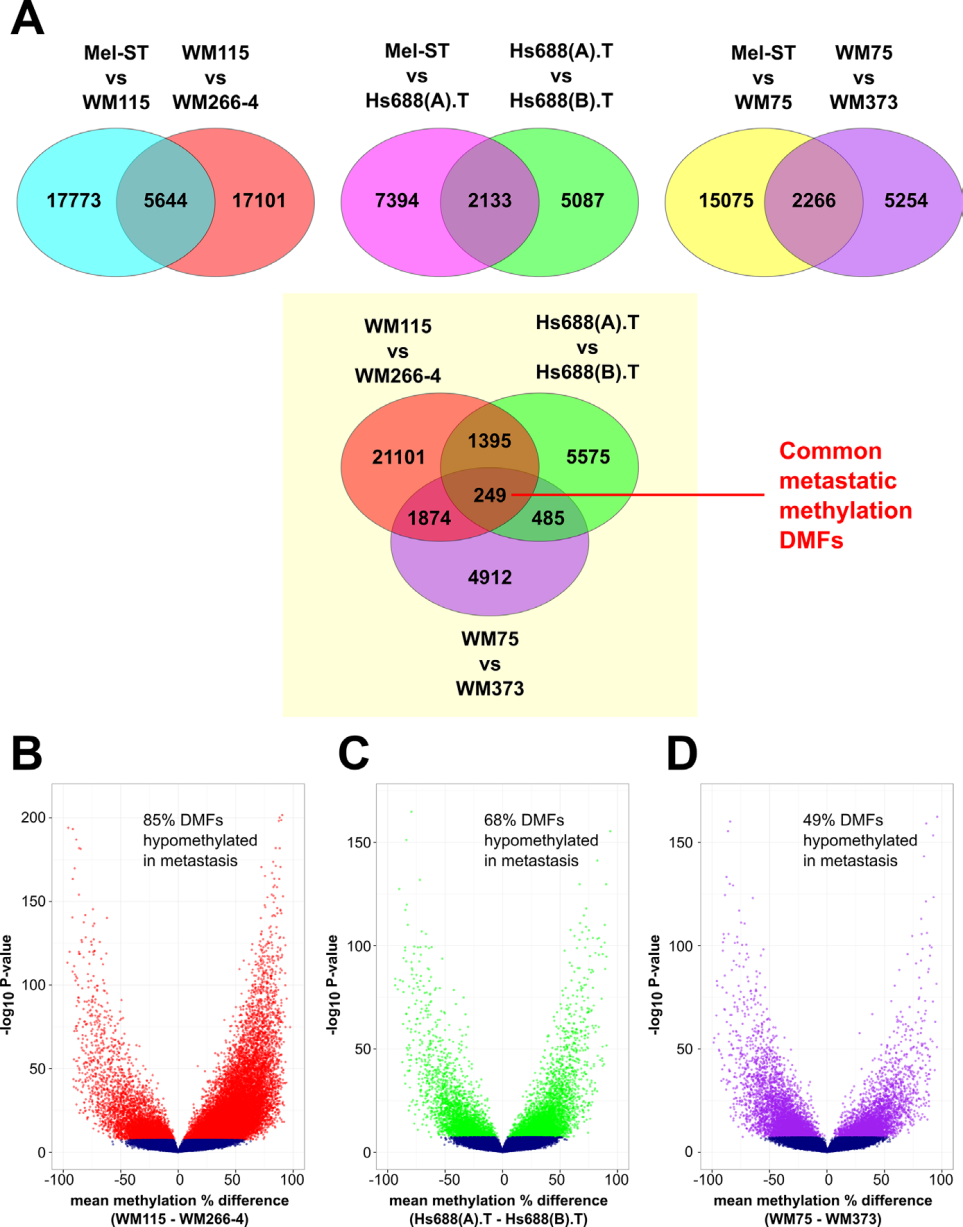
Strategy and landscape of differential methylation in melanoma metastasis (**A**) Details of the differential methylation analysis. The comparisons between the cell lines are written on the top of each Venn diagram and the number indicates the differentially methylated fragments (DMFs) in each of these comparisons. (**B**–**D**) Volcano plots showing the methylation changes between each metastatic cell line compared to the matched primary cell line. The mean methylation changes for the analysed fragments between the pairs are represented on the x-axis. Blue indicates fragments that were not significant. The DMFs were represented by red (for WM115 vs. WM266-4), green (for Hs688(A).T vs. Hs688(B).T) and purple (for WM75 vs. WM373). The y-axis shows the -log10 of the *P*-values.

It is common for DNA methylation studies to collectively compare differences in DNA methylation between two groups of samples (such as primary and metastatic tumors). However, because the 3 pairs of cell lines each contained distinct epigenomes (Figure 1), we performed differential methylation analysis on each cell line pair independently (Figure 2A). We used *Msp*I fragments (40–220 bp) as the unit of analysis rather than individual CpG sites or a tiled window approach, as we have described previously [19–21]. Fragments were analysed where 10 or more reads were obtained in at least two CpG sites in each sample. The fragments that passed statistical significance (Fisher's exact test followed by Bonferroni correction), and which had ≥ 25% mean methylation difference, were considered to be differentially methylated fragments (DMFs). We identified 23417, 9527 and 17341 differentially methylated fragments (DMFs) between Mel-ST and the primary melanoma cell lines (WM115, Hs688(A).T and WM75), respectively (detailed data and statistical analyses are given in Supplementary Table S5).

In all three cases, we found primary melanoma cell lines were overall hypomethylated compared to normal melanocytes (Supplementary Table S5). We also identified 22745, 7220 and 7520 DMFs between the primary melanoma cell lines (WM115, Hs688(A).T, WM75) and the corresponding matched metastatic cell lines, respectively. Two metastatic cell lines were strikingly more hypomethylated than the primary melanoma cell lines in each pair, while approximately half the DMFs in WM373 were hypomethylated relative to WM75 in the third pair (Figure 2B–2D and Supplementary Table S5). A total of 5644, 2133 and 2266 DMFs showed changes in methylation between Mel-ST and the primary melanoma cell lines, and the same DMFs also showed changes between the primary melanoma and the corresponding metastatic melanoma cell lines (Figure 2A). This subset of DMFs comprised ~1/3 of all the DMFs identified between the primary melanoma and metastatic melanoma cell lines, and the changes observed in each DMF corresponded to one of four patterns of methylation change, as illustrated in Supplementary Figures S3–S5.

### Validation of DMF containing genes

**Figure 3:**
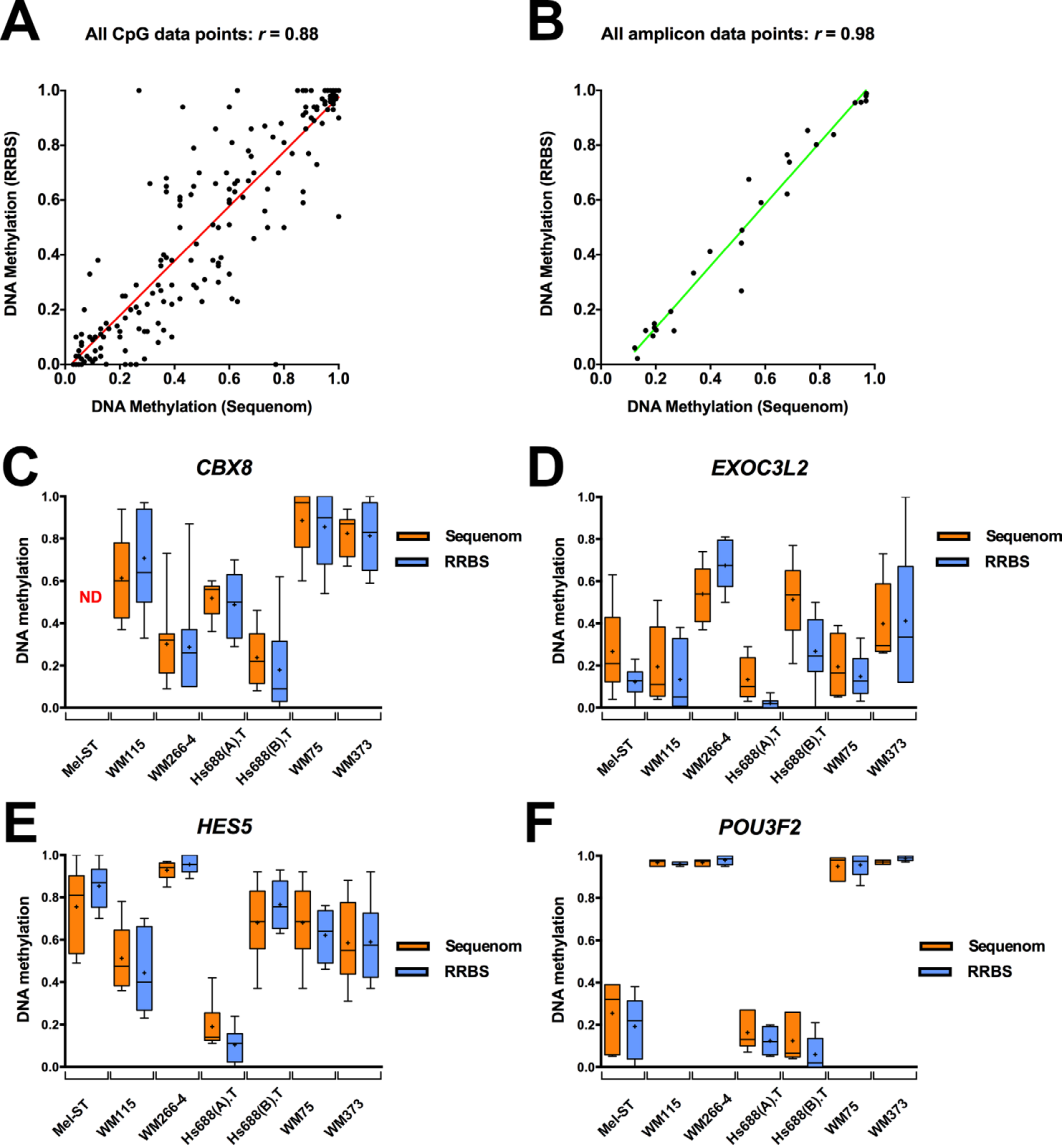
Validation of RRBS DNA methylation data using Sequenom (**A**) Correlation of DNA methylation profiles at individual CpG sites covered by both techniques (RRBS and Sequenom) in the analysed cell lines. (**B**) Correlation of mean methylation of the fragments covered by both techniques (common amplicon) in the cell lines. (**C–F**) Comparative RRBS and Sequenom methylation profiles of four genes (*CBX8, EXOC3L2, HES5, POU3F2*) investigated in the analysed cell lines. Three genes (*HES5*, *CBX8* and *EXOC3L2*) were significantly differentially methylated in two or more primary and metastatic cell line pairs, while the fourth gene, *POU3F2*, exhibited a different promoter methylation pattern across the pairs of cell lines. The box represents the 25^th^ and 75^th^ percentiles and the whiskers are drawn to the minimum and maximum values, and “+” denoted the mean methylation over the common amplicon. For CBX8, the Mel-ST methylation profile could not be confidently determined due to low coverage.

We chose four genes with their associated DMFs (*CBX8, EXOC3L2, HES5*, and *POU3F2*) as representative examples to demonstrate that the results from RRBS could be validated in mass spectrometry-based Sequenom analysis (EpiTYPER) [22]. These regions were selected because at least two cell line pairs showed differential methylation in these regions in RRBS, the regions harbored multiple RRBS fragments, they had good sequencing coverage, and a suitable Sequenom assay to compare methylation status was able to be developed for these regions. In total, for these four genes, 107 CpG sites were included in the Sequenom assays (Supplementary Table S6–S7). Methylation percentages determined at each CpG site by RRBS and Sequenom were highly concordant (Pearson's *r* = 0.88, two-tailed test, *P*-value = 0.0001, Figure 3A), and they were further improved by performing comparisons over commonly analysed amplicons (Pearson's *r* = 0.98, Figure 3B and Table 1). RRBS and Sequenom were also concordant in Bland-Altman (BA) analysis (Supplementary Figures S6–S7). (*P*-value < 0.05, Figure 3C–3F) with very low bias (standard deviation ranging from 0.072 to 0.252) between RRBS and Sequenom (Table 1). Taken together, these results suggested that there was high technical reproducibility of the RRBS data described here.

**Table 1:** Summary of RRBS validation of epigenetic changes in four genes using sequenom

		CpG correlation (Sequenom-RRBS)		Bland Altman analysis		
Nearest Gene	Feature	*r*-value	*P*-value	Bias	SD of bias	Limits of agreement
*CBX8*	Promoter	0.86	< 0.0001	−0.0087	0.16	From −0.33 to 0.31
*HES5*	Promoter, CGI core	0.87	< 0.0001	2.4e-005	0.15	From −0.29 to 0.29
*EXOC3L2*	Promoter	0.50	0.0008	−0.066	0.24	From −0.54 to 0.41
*POU3F2*	Promoter, CGI core	0.99	< 0.0001	−0.019	0.07	From −0.15 to 0.11
All data points (CpG site)	NA	0.88	< 0.0001	−0.022	0.16	From −0.35 to 0.31
All data points (amplicon)	NA	0.98	< 0.0001	−0.0251	0.08	From −0.18 to 0.13

### Identification of shared methylation changes in melanoma metastasis

Shared methylation changes between primary and metastatic cell lines were identified from the lists of DMFs. The percentage of overlap of DMFs between any two cell-line pairs varied from 10.2% to 28.2% (Supplementary Table S8; the patterns of DNA methylation in these common fragments are shown in Supplementary Figures S8–S10). Overall from 47% to 72% of the shared DMFs changed methylation in the same direction between any two cell-line pairs (Supplementary Table S9), with a total of 249 DMFs being shared between all three primary-metastatic pairs (Figure 2A).

### Further characterization of shared metastasis-related DMFs

**Figure 4:**
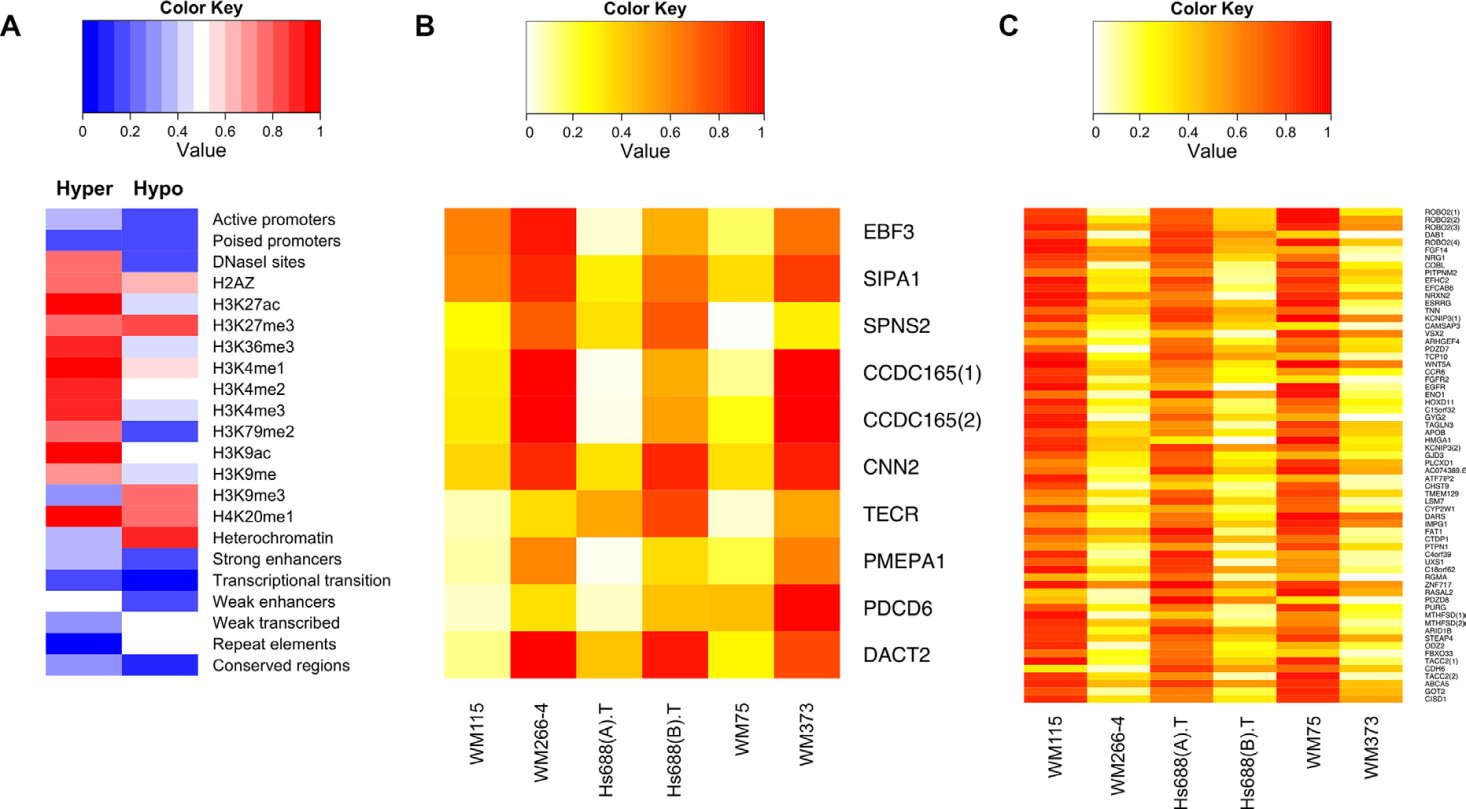
Genome regulatory feature and methylation status of the common driver hyper and hypo methylated DMFs in melanoma metastasis (A) The percentage overlap of the common hyper- and hypomethylated DMFs with a genome feature (using publicly available data from ENCODE) is calculated in a scale of 0 to 1 and plotted as a heatmap. (B) Methylation of the primary and metastatic cell lines in the common hypermethylated DMFs (C) Methylation of the primary and metastatic cell lines in the common hypomethylated DMFs is shown. Low (= 0) to high (= 1) methylation is shown as a continuous variable from white to red.

Of the 249 shared metastatic DMFs, 65 were hypomethylated (associated with 59 genes) and 10 were hypermethylated (associated with 9 genes) in each metastatic cell line versus the matched primary melanoma cell line (Supplementary Data Files S1 and S2). Regulatory features in the context of the chromatin state map and genome regulation were identified in the DMFs using publicly available data from ENCODE, aggregating the data from nine cell lines [23]. DNase hypersensitive regions, active histone modification marks (such as H3K27ac, H3K36me3, H3K9ac) and enhancers were more abundant, while repressive histone marks (such as H3K27me3 and H3K9me3) were less abundant in hypermethylated versus hypomethylated DMFs (Figure 4A). In contrast, weakly transcribed and heterochromatic genomic regions were more frequently associated with hypomethylated DMFs (Figure 4A and Supplementary Table S10).

Shared hypermethylated DMFs (shown in Figure 4B) were not identified in this study within repeat elements, eg hypermethylation of LINE elements, as others have reported in the peripheral blood of metastatic melanoma patients [24], but 31% of shared hypomethylated DMFs were located in SINE elements, and a total of 48% of the shared hypomethylated DMFs were located within repeats (Supplementary Table S10). With respect to transcription factor binding sites, RNA Polymerase II subunit sites were enriched in shared hypomethylated DMFs, while GA-binding protein *(GABP)* and TATA box binding factor (*TAF1)* sites were enriched in hypermethylated DMFs (Supplementary Table S11).

Eight of 10 hypermethylated DMFs were contained in introns of protein-coding genes. Hypermethylation of the promoter region, 993 bp upstream from the transcription start site (TSS), was observed for early B cell factor 3 (*EBF3*) in all metastatic cell lines. Suppressor of glucose autophagy associated 2 (*CCDC165* or *SOGA2*) contained two adjacent hypermethylated DMFs in the first intron (within 1kb from the TSS). Both *EBF3* and *SOGA2* DMFs were situated within core CpG islands. Functional enrichment analysis of the genes that contained hypermethylated DMFs (in promoters or gene bodies) indicates that they were mainly involved in cellular organisation, intracellular signalling and transcriptional regulation (Supplementary Table S12). Forty five percent of the hypomethylated metastatic DMFs were located in gene bodies, with the majority being in introns. Genes encoding high-mobility group protein A1 (*HMGA1*), Kv Channel Interacting Protein 3 (*KCNIP3*) and Apolipoprotein B (*APOB*) shared promoter hypomethylation in all metastatic melanoma cell lines. *HOXD11* was consistently hypomethylated, while *ROBO2* shared four intronic hypomethylated DMFs in all metastatic cell lines (gene promoter- and gene body-associated hypomethylated DMFs are shown in Figure 4C). Differential methylation of *HOX* family genes has previously been reported between brain and lymph node metastasis [14]. Interestingly, 50% of the hypomethylated DMFs were located in CpG island shores (Supplementary Data File S1). Promoter- or gene body hypomethylated DMFs were significantly enriched in the regulation of cell differentiation, motility and adhesion and were related to cancer pathways (Supplementary Table S13, P < 0.05, Fishers exact test).

### Validation of genes associated with shared DMFs using TCGA melanoma patients

Validation of the hyper and hypomethylated DMFs was carried out using The Cancer Genome Atlas (TCGA)-SKCM (melanoma) dataset, which contains 450K-DNA methylation microarray data for 458 patients (99 primary and 359 metastatic tumors). The CpG sites in the 450K-microarray platform are unevenly distributed in the genome, being mainly contained within promoter regions. For a comparable analysis, we analysed individual CpGs from TCGA 450K data that directly overlapped with, or were adjacent to our identified DMFs (i.e. CpGs within 500 bp upstream or downstream of the DMF) in primary and metastatic melanoma patients. For 26 (i.e. for 5 hypermethylated DMFs and for 21 hypomethylated DMFs) out of the 75 common DMFs identified in RRBS, we found a comparable CpG site that directly overlapped or was adjacent in the TCGA dataset. Similar to the RRBS analysis, significant hypermethylation in metastatic patients was confirmed for three genes (*EBF3*, *SIPA1* and *TECR*) in TCGA. On the other hand, three genes (*EFCAB6*, *ESRRG* and *AC074389.6)* were significantly hypomethylated in metastatic patients (*P*-values ranged from 0 to 0.0421, Mann Whitney *U* test, Supplementary Tables S14–S15), resembling our RRBS analysis. For AC074389.6, the associated DMF was 22 kb upstream from the transcription start site, while for *EFCAB6*, *ESRRG*, *SIPA1* and *TECR* the associated DMFs were located within the introns of the genes. Although intriguing, it is presently less clear how intergenic and gene-body methylation patterns would be related to corresponding changes in mRNA levels in cancer.

**Figure 5:**
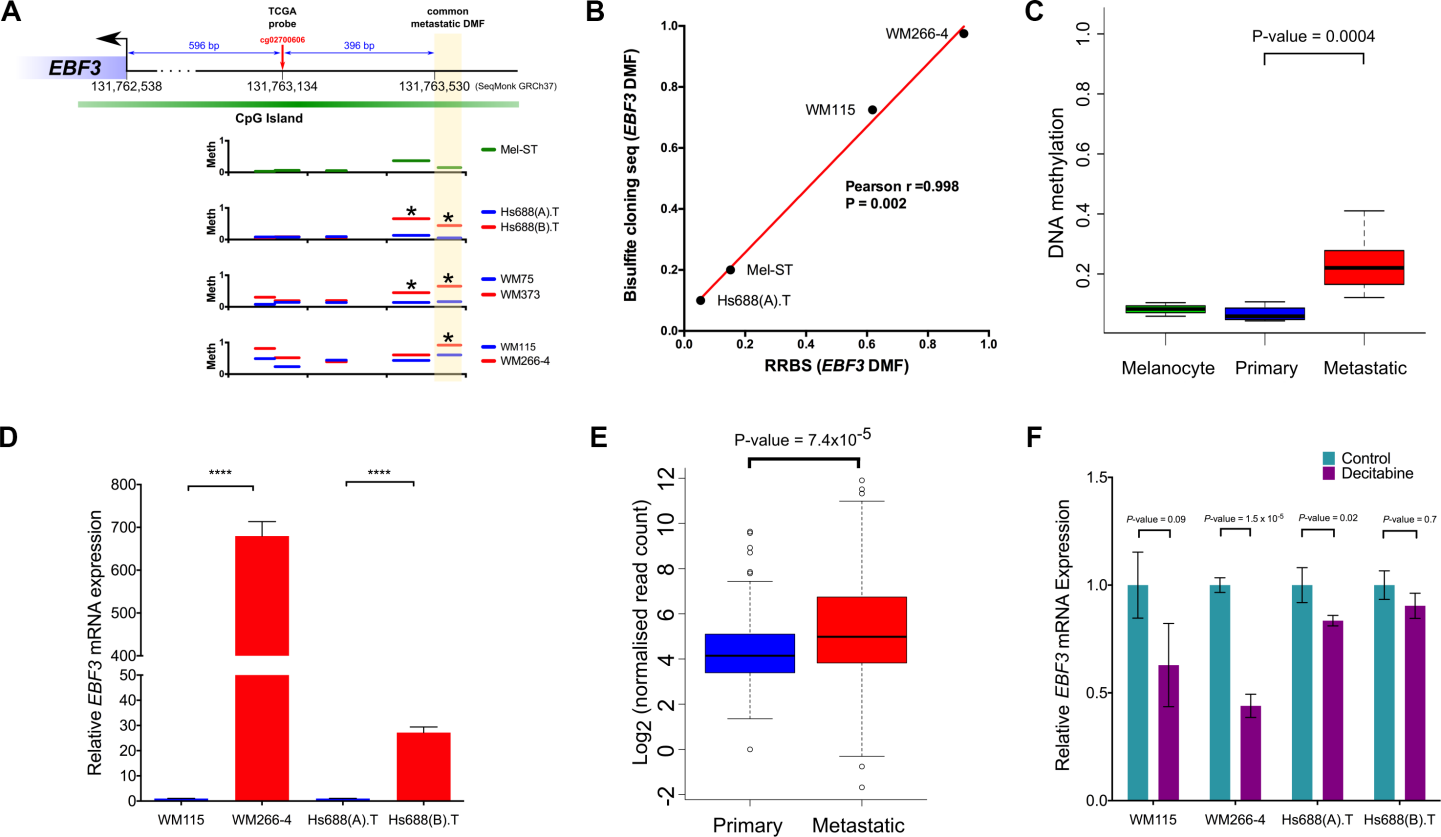
Confirmation of EBF3 promoter methylation using bisulfite sequencing, validation in an independent cohort, analysis of mRNA expression levels and the effect of DNA methylation inhibitor treatment on EBF3 (**A**) Promoter methylation map of *EBF3*. The genomic coordinates generated using DMAP tools were obtained from SeqMonk feature table information (GRCh37 version). Coordinates for *EBF3* were 131633547-131762538 (antisense). (**B**) Correlation of DNA methylation status in the DMF region of *EBF3* (-993 bp) between RRBS and locus-specific bisulfite sequencing. The x and y axes represent the degree of methylation in the RRBS versus locus-specific bisulfite sequencing (on a scale of 0 to 1.0) and the red line shows the regression line. Locus-specific bisulfite sequencing methylation data from multiple clones were averaged for the four cell lines to compare with RRBS data. (**C**) Box plots representing DNA methylation in 3 melanocyte samples, 4 primary melanoma and 33 metastatic melanoma samples (obtained from GSE44661). Metastatic melanomas contained higher *EBF3* promoter methylation (*P*-value = 0.0004, Mann Whitney *U* test). (**D**) *EBF3* expression levels in metastatic cell lines (hypermethylated) compared to their corresponding primary melanoma cell lines (hypomethylated). **** *P*-value < 0.0001, Welch's *t* test). *EBF3* is completely silenced in the Mel-ST cell line and a Ct value could not be detected for this cell line. (**E**) Box plots representing the distribution of *EBF3* mRNA expression in 99 primary melanomas and 359 metastatic melanoma patients (from TCGA data). Metastatic melanomas expressed significantly higher *EBF3* levels (*P*-value= 7.427e–05, Mann Whitney *U* test). (**F**) *EBF3* expression levels before and after 5-Aza-2′-deoxycytidine treatment (72 h). Mel-ST was not included in this figure, as *EBF3* expression was not detected before and after treatment. The error bar in panel c and e represents Mean ± standard error of mean.

For *EBF3*, the hypermethylation was identified in the promoter, 993 bp upstream from the transcription start site and was strongly validated in the TCGA patients (*P*-value = 0.0015, Mann Whitney *U* test; the location of the TCGA CpG is shown in Figure 5A). In contrast to the intergenic and gene-body methylation, promoter associated methylation changes provide a well-documented mechanism of altering gene expression, by potentially altering transcription factor binding to gene promoters.

The CpG site at -993 bp, analysed in the TCGA data, was also investigated in the seven cell lines (melanocyte and primary and metastatic melanomas). The methylation profiles of the CpGs in commonly analysed DMFs, located in the *EBF3* promoter for the seven cell lines, are shown in Figure 5A. Overall, the methylation patterns indicated a localized change in the identified promoter region, and in the adjacent fragments. Other fragments in the promoter did not exhibit a significant difference (Figure 5A).

### Confirmation of *EBF3* promoter hypermethylation using bisulfite sequencing

To confirm the RRBS results, which showed hypermethylated fragments in the *EBF3* promoter in metastatic melanoma cell lines, we performed locus specific bisulfite cloning and sequencing in four cell lines (Mel-ST, WM115, WM266-4 and Hs688(A).T). The results confirmed the *EBF3* promoter DMF hypermethylation in the metastatic melanoma cell lines, and we observed excellent concordance for the DMF methylation between RRBS and the bisulfite sequencing (Pearson's *r* = 0.998, *P*-value = 0.002, Figure 5B).

### Validation of *EBF3* promoter hypermethylation in an independent melanoma cohort

Additional support for *EBF3* promoter hypermethylation in metastatic versus primary melanomas was obtained from *EBF3* promoter methylation analysis in a second independent melanoma cohort, consisting of 450K methylation data for three normal melanocytes, four primary melanomas and 33 metastatic melanomas [14]. In this cohort *EBF3* promoter methylation in metastatic melanomas (median = 0.22) was significantly greater than promoter methylation of primary melanomas or normal melanocytes (median= 0.08 and 0.06 respectively, *P*-value = 0.0004, Mann Whitney *U* test, Figure 5C). Therefore, we next decided to investigate the functional role of *EBF3* as a potential driver of melanoma metastasis.

### *EBF3* promoter methylation was associated with increased *EBF3* expression in melanoma cell lines and in TCGA patients

To determine whether *EBF3* promoter hypermethylation influenced *EBF3* expression we quantified *EBF3* mRNA expression levels in two paired cell lines (WM115, Hs688(A).T, and WM266-4, Hs688(B).T). Unfortunately, the third cell line pair (WM75 and WM373) was extremely slow growing, and as a consequence no further analysis (including RNA extraction) could be carried out using this cell line pair. Surprisingly, *EBF3* mRNA levels were significantly higher in the two metastatic versus the primary cell lines (Figure 5D). (WM266-4 versus WM115 was 620-fold higher, *P-value* = 6.5e–14; Hs688(B).T versus Hs688(A).T was 29.5-fold higher, *P*-value = 8.5e–16). The observation that the WM266-4 promoter was relatively more highly methylated compared to the other cell lines (see Figure 4) may be related to the 620-fold higher expression of *EBF3* than WM115 in this cell line. To determine whether the cell line expression data was reflected in the findings from melanoma patient samples, we compared *EBF3* mRNA expression in primary and metastatic patients from TCGA RNA-Seq data. Mirroring the cell lines, significantly higher expression of *EBF3* mRNA (*P*-value = 7.427e–05, Mann Whitney *U* test, Figure 5E) was observed in metastatic tumors (median normalized count = 31.63, mean count = 156) compared to primary melanomas (median normalized count = 17.87, mean = 55.16). *EBF3* promoter hypermethylation and corresponding gene expression were positively correlated in TCGA melanoma samples (*P-value* = 0.025, Spearman's rank correlation). Taken together, these results suggest that high *EBF3* promoter methylation is associated with higher levels of *EBF3* expression in metastatic melanomas.

### *EBF3* promoter methylation was reversed by methylation inhibition

To determine whether demethylation of *EBF3* following DNA methylation inhibitor treatment leads to altered *EBF3* mRNA levels, we treated the cell lines with 5-Aza-2′-deoxycytidine (also known as decitabine), which inhibits DNA methylation in a replication-dependent manner. Following decitabine treatment, we confirmed demethylation of the DMF region in the *EBF3* promoter (see Supplementary Figure S11) and measured *EBF3* mRNA expression levels 72 h post treatment. Consistent with our prediction, we observed decreased methylation of the DMF region in the *EBF3* promoter, and significantly reduced *EBF3* mRNA levels as result of the demethylation in WM-266-4 and Hs688(A).T cell lines (Figure 5F). Further, although not statistically significant (*P*-Value = 0.09, Welch *t* test), the WM115 cell line also showed a reduction (37.1%) in mRNA level (Figure 5F). We did not observe noticeable changes in Hs688(B).T. However, this cell line replicates slowly and it is likely that 72 h was insufficient to induce demethylation. Decitabine treatment was unable to alter *EBF3* expression in Mel-ST cells. This was expected as our data show that the *EBF3* promoter was already unmethylated in these cells, and suggests that the reduction of *EBF3* mRNA levels in the melanoma cell lines is less likely to be a non-specific effect.

### *EBF3* expression promoted aggressive phenotypic features in melanoma cell lines

**Figure 6:**
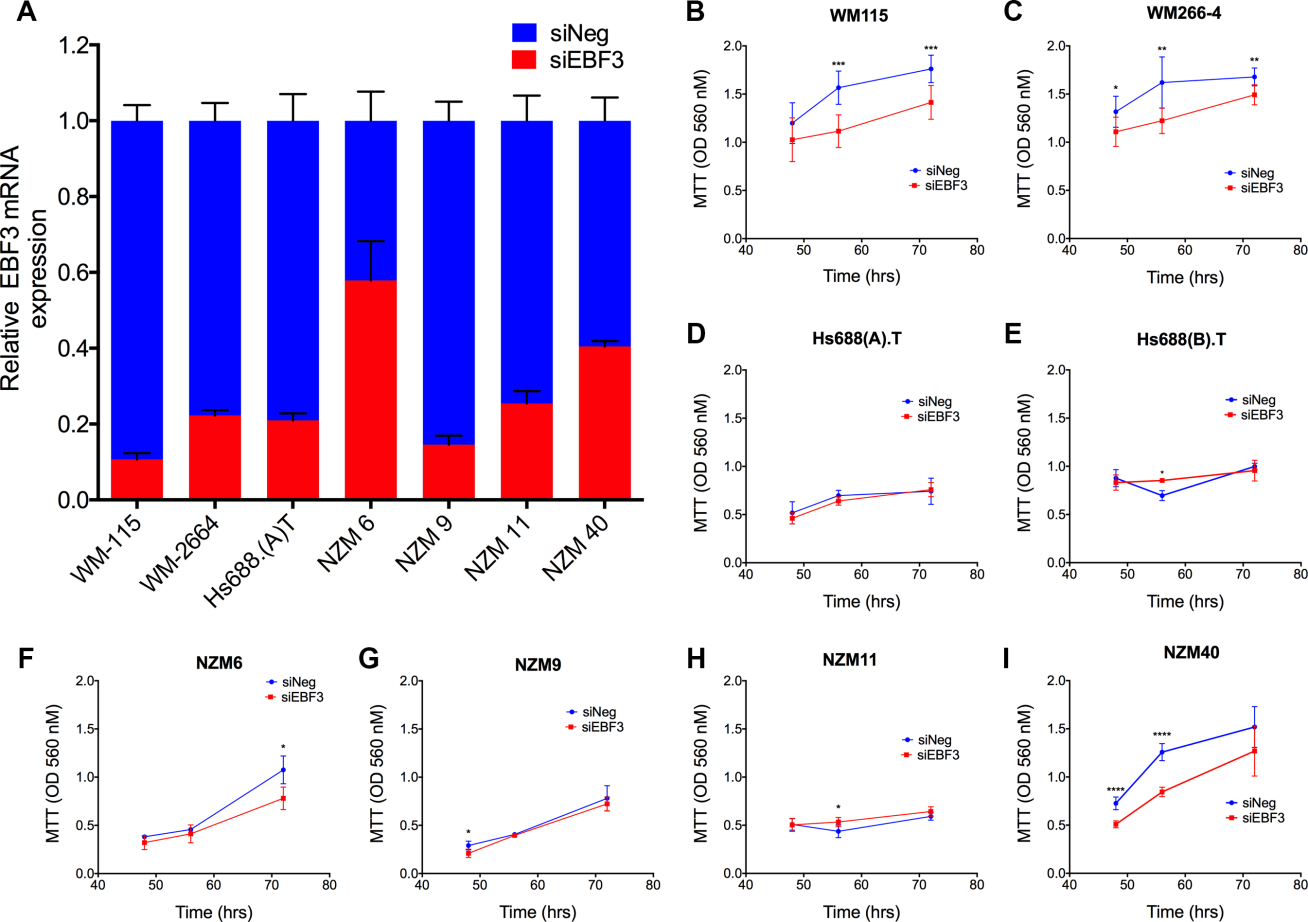
siRNA knockdown efficiencies and MTT assays for eight melanoma cell lines before and after EBF3 knockdown (**A**) Relative expression of *EBF3* mRNA levels as determined by quantitative real-time PCR from seven melanoma cell lines transfected with either siNeg or siEBF3. Error bars represent the standard error of the mean. Delta CT values were normalized to mRNA expressions of house keeping genes *RPL27* and *SRP14*. SiRNA knockdowns reduced the EBF3 protein level (see Supplementary Figure S12 for representative Western blot), and an efficient knockdown of *EBF3* mRNA was observed in the majority of cell lines from 48 h until 96 h. (**B**–**I**) MTT assays before and after *EBF3* knockdown. These experiments were performed over 48, 72 and 96 h in the presence of siEBF3 and siNEG. However, for the Hs688(A).T and Hs688(B).T) cell line pair we observed that the 96 well plates became over-confluent at 96 h, resulting in inaccurate proliferation measurements (this was due to the large physical size of these cells). Therefore for these two cell lines the proliferation was measured at 48, 56 and 72 h after siEBF3 transfection. Values (y-axis) are representative of optical density (OD) values measured at 560 nm ± standard deviations. Statistical significance was determined using Welch's *t* test. *P < 0.05; **P < 0.01, ***P < 0.001. The error bars indicate 95% confidence intervals.

To investigate whether the elevated *EBF3* expression was functionally significant in melanoma cells, we knocked down *EBF3* using siRNAs in matched primary/metastatic cell lines (WM115, Hs688(A).T, WM266-4, Hs688(B).T) and in four additional NZM metastatic melanoma cell lines (NZM6, NZM9 NZM11, NZM40), which were included to facilitate the investigations of *EBF3* function. The metastatic NZM melanoma cell lines were comprised of both invasive and non-invasive cell lines, which we have characterised previously [25]. Hs688(B).T, NZM6 and NZM11 were non-invasive in Boyden chamber assays, whereas WM115, WM266-4, Hs688(A).T, NZM9, and NZM40 were invasive metastatic melanoma cell lines [25]. An siRNA pool containing four siRNAs against *EBF3* resulted in 75% to 90% knockdown of *EBF3* mRNA, except in NZM6 and NZM40 where the knockdown efficiencies were 42% and 60% respectively (Figure 6A). Efficient knockdowns were observed in cell lines until 96 h post-transfection, as assessed using RT-QPCR, although knockdown was maintained in Hs688(A).T at 72 h, but was lost at 96 h post-transfection. Knockdown of EBF3 protein was confirmed in two of the cell lines by Western blot (Supplementary Figure S12). The mean viability for the *EBF3* siRNA (siEBF3) treated cells ranged from 86.5% to 95.2% as determined by trypan blue staining 72 h following *EBF3* knockdown, while the mean viability for control siRNA treated cells (siNeg) ranged from 88.5% to 95%, and viability continued to be high at 96 h following knockdown. Cell viability data were also confirmed by caspase-3 assays (data not shown). Overall, these data suggest that reduction in MTT values following *EBF3* knockdown were not due to cell death, but rather were due to inhibition of total cell mass, for example through inhibiting proliferation (Supplementary Figure S13). Significantly reduced MTT values were observed following *EBF3* knockdown in MTT assays in WM115, WM266-4, NZM40 and NZM6 cells at 48 h and 72 h, respectively post-siRNA treatment (*P*-value = 0.0001 and 0.001 at 72 h and 96 h, *P*-value = 0.019, 0.002 and 0.002 at 48 h, 72 h and 96 h, *P*-value = 6.4e–06 and 1.9e–07 at 48 h and 72 h, and *P*-value = 0.03 at 96 h, Welch *t* test, Figure 6B–6I).

**Figure 7:**
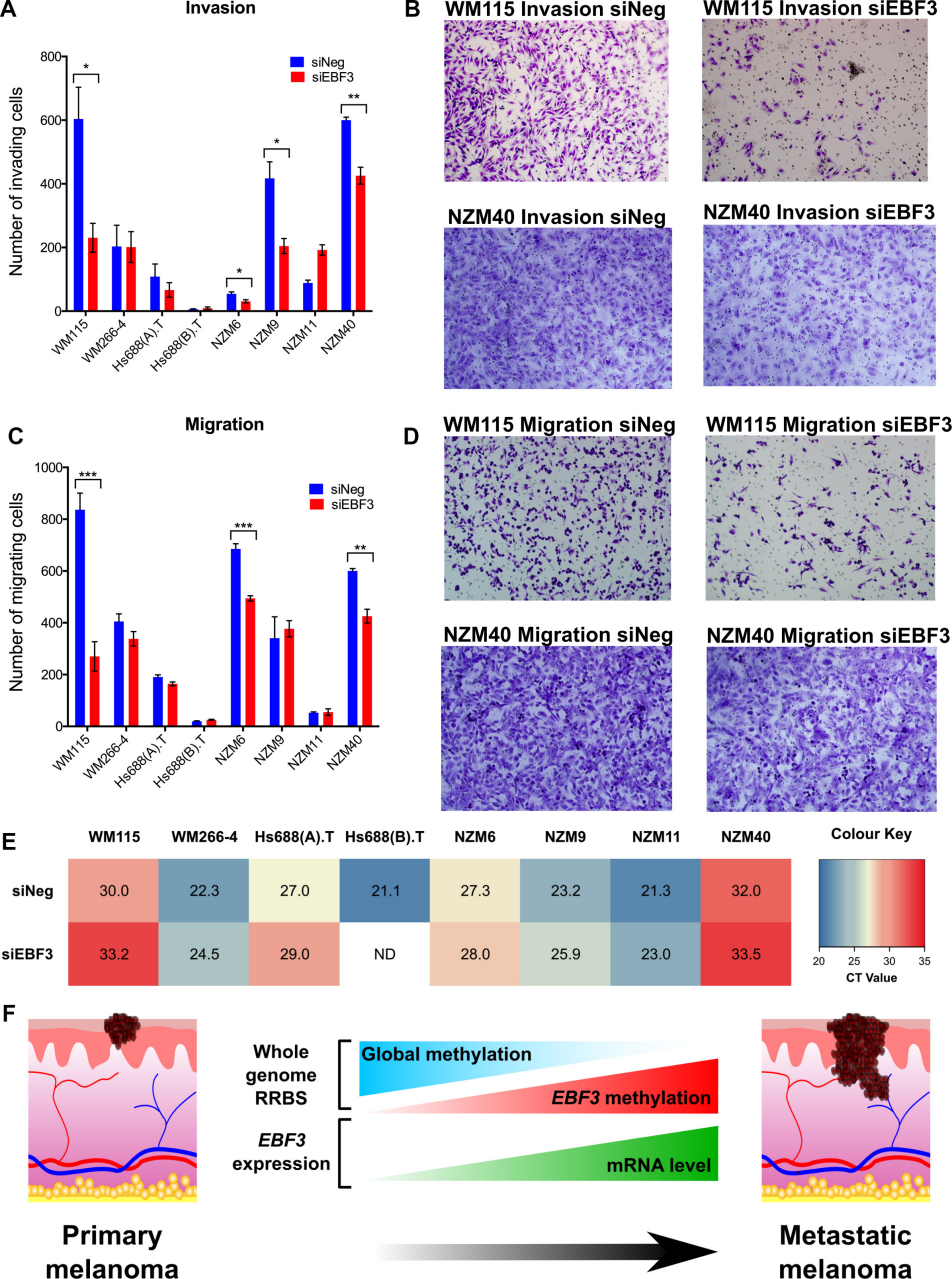
EBF3 expression influences invasive and migratory properties of melanoma cells (**A**) A Matrigel insert (Boyden chamber assay) was used to assess differences in the numbers of invading cells (y-axis) in eight cell lines transfected with siEBF3 or siNeg (48 h post siRNA transfection). (**B**) Representative images of differences in the amount of cells demonstrating invasion for cell lines WM115 and NZM40. Fewer invading cells can be observed in siEBF3 treated cells (48 h post siRNA transfection). (**C**) The number of migratory cells in eight cell lines (y-axis), assessed by the Boyden chamber assay without Matrigel. (**D**) Representative images of the differences in the number of migrating cells in siEBF3 and siNeg conditions for WM115 and NZM40. (**E**) A heatmap demonstrating the CT values of *EBF3* expression in the eight cell lines, as determined by qPCR (normalized to the housekeeping genes *RPL27* and *SPR14*). The values indicate baseline *EBF3* expression (siNeg) and expression following knockdown (KD), (siEBF3). ND indicates there is no data for *EBF3* expression following KD for Hs688(B).T, as it was an extremely slow growing cell line and insufficient cells could be obtained for this experiment. Significance was determined using Welch's *t* test. (F) Cartoon summarizing the mechanistic role of DNA methylation and *EBF3* expression changes we identified associated with progression from primary to metastatic melanoma. *P < 0.05; **P < 0.01, ***P < 0.001. Error bars in Figures a and c indicate means ± standard error of the mean.

We next determined whether *EBF3* knockdown affects melanoma cell migration and invasion using Boyden Chamber assays, with or without a Matrigel membrane coating, to investigate invasion and migration, respectively. Depletion of *EBF3* resulted in significant reduction in migration in three of eight cell lines (WM115, NZM6 and NZM40) at 48 h (3.1-fold, 1.4-fold, and 1.4-fold decrease, *P-value*s = 0.0002, 0.0001, and 0.002, respectively, Figure 7C–7D). Note that NZM6 is a non-invasive cell line, but was able to migrate across a porous membrane, and the knockdown of *EBF3* prevented this migration. Similarly, following *EBF3* knockdown, significantly reduced invasion was observed in three cell lines (WM115, NZM9 and NZM40) at 48 h following *EBF3* depletion (2.6-fold, 2-fold and 1.4-fold decrease, *P*-values = 0.0160, 012 and 0.002, respectively, Figure 7A–7B). *EBF3* real-time PCR data (i.e. CT values) were positively correlated with the fold-reduction in migration or invasion (Spearman rho= 0.656, *P*-value = 0.01, Supplementary Figure S14). Taken together, these data suggest that *EBF3* expression is associated with aggressive phenotypic behavior in metastatic melanoma cell lines (Figure 7E). Despite the observed changes in migration and invasion, we did not observe changes in the expression of EMT markers (*SNAIL, SLUG, TWIST, ZEB1, ZEB2, WNT5A*) by real-time PCR in the melanoma cell lines following *EBF3* knockdown (Supplementary Figure S15A– S15E).

## DISCUSSION

We have carried out, to our knowledge, the first sequencing-based genome-wide DNA methylation study of matched primary and metastatic cell lines, as a means to identify candidate epigenetic drivers of melanoma metastasis. The primary aim of our study was to identify candidate “driver” DNA methylation changes, using pairs of matched primary and metastatic melanoma cell lines, focusing on metastasis-related changes, while at the same time minimizing the detection of random epigenetic differences resulting from inter-patient or intra-tumoral cellular heterogeneity.

In a similar approach, using 450K-DNA methylation arrays to analyse multiple cancer types (melanoma, breast cancer and colorectal cancer), shared “driver” epigenetic differences were identified between 3 pairs of metastatic and primary cancer cell lines. In metastatic tumors, site-specific hypomethylation was associated with reactivation of a cryptic transcript of TBC1 Domain Family Member 16 (*TBC1D16*) and poor clinical outcome [15]. However, none of the CpG sites that were identified as significant in that study between primary and metastatic tumors, were observed in our analysis.

A framework for identifying putative driver, and passenger epigenetic changes has not yet been established [6]. In the present work we devised a framework whereby epigenetic changes were identified primarily as shared DMFs that showed a similar direction of DNA methylation change in all metastatic samples. Following this, we analysed the methylation status of one or more CpGs located in, or closely associated with the shared common DMF in additional cohorts of melanomas to determine whether a more generalized change could be observed in a significant proportion of metastatic melanomas versus primary melanomas. Candidate genes associated with the identified DMFs were then validated using functional assays in melanoma cell lines to determine whether the identified DMF could potentially represent an epigenetic driver of metastasis (“epi-driver” versus “epi-passenger”).

Conceptually, epi-drivers could be identified using two approaches; (i) comparing the frequency of commonly shared DNA methylation alterations of genes/regions with other genes/regions in multiple samples, and (ii) measuring effect sizes or degree of epigenetic changes to determine the functional role of each epigenetic change. Similar to frequency-based approaches used to identify cancer driver mutations [6], we hypothesize that regions that are frequently epigenetically altered, and shared in multiple samples, are more likely to be functionally relevant, and combining the two approaches would allow frequently shared epigenetic driver DNA methylation changes to be evaluated.

RRBS analysis greatly expands the characterization of global methylation patterns in melanoma, as compared to array-based platforms. In the latter, predominantly promoter methylation changes are characterized [11, 14, 15], whereas in RRBS, in addition to promoter methylation, both gene body and intergenic methylation are strongly represented. The paradigm of global hypomethylation in primary cancers relative to pre-cancerous cells [9] was replicated in our study, but we also found that in at least two of the cell line pairs, melanoma cells underwent a second wave of global hypomethylation, and site-specific gains of methylation, upon metastasis. Hypomethylated DMFs were enriched for repetitive elements, particularly SINE elements, and contained repressive histone marks in our chromatin map analysis, which has significant implications for understanding epigenetic mechanisms in cancer metastasis; hypomethylation mediated re-activation of transposable elements, and elevated expression of oncogenes as a result of promoter hypomethylation [9], might induce genomic damage and chromosomal instability, making cells prone to structural rearrangements [26]. Gene mutations were variably distributed between the three cell line pairs, and are thought to have contributed to the divergence between methylomes, and possibly also to the functional characteristics of some of the cell lines. Recent studies report that mutations in epigenetic modifier genes, such as *DNMT*, *TET* family genes, or *EZH2*, lead to epigenomic and trancriptomic alterations that differ from tumors without these mutations [27].

The promoter of *EBF3* was hypermethylated in all three metastatic melanoma cell lines. An adjacent *EBF3* promoter region, approximately 200–300 bp upstream from the region identified by RRBS, was investigated in TCGA melanoma data, and was also found to exhibit high levels of DNA methylation. Promoter-specific hypermethylation is associated with tumor suppressor gene silencing in cancer [9], and has been reported previously in several gene-specific melanoma methylation studies [3, 12, 13, 28, 29].

*EBF3* promoter hypermethylation in the metastatic melanoma cell lines was unexpectedly associated with elevated (rather than decreased) *EBF3* expression, and this was also observed in melanoma patients in the TCGA database. Analysis of TCGA data suggested that *EBF3* was relatively highly expressed in a subgroup of metastatic melanomas as compared to primary melanomas. *EBF3* plays an important role in cell lineage commitment, migration and differentiation of several cell types, including B-lymphocytes and neurons [30, 31]. Hypermethylation of the *EBF3* promoter (at a region between +700 and +1030 downstream from the transcription start) and down-regulation of *EBF3* expression in contrast to up-regulation, as observed in our study, has been reported in several cancers [32–34]. Therefore *EBF3* has previously been postulated to be a tumor suppressor gene [31]. In contrast to the downstream promoter regions that were investigated previously, we investigated a CpG rich region upstream of the transcription start site (i.e. different region from that previously investigated), which together with our complementary observations, suggests that *EBF3* may have oncogenic tumor promoting properties associated with melanoma metastasis. An oncogenic role for *EBF3* has been reported in several other cancer types, including elevated expression of *EBF3* in phaeochromocytoma. In addition, forced expression of *EBF3* in medulloblastoma cancer stem cells, or in HepG2 hepatocellular carcinoma cells was associated with enhanced tumorigenesis, or increased cell cycling, respectively [35–37].

When melanoma cell lines were treated with a demethylating agent, decitabine, reduction in *EBF3* gene expression was associated with demethylation of the *EBF3* promoter DMF, supporting the notion that reducing methylation of the *EBF3* promoter causes reduction of the corresponding *EBF3* mRNA levels.

*EBF3* knockdown in our functional studies was associated with reduced MTT values, migration and/or invasion in half (4 of the 8) of the metastatic melanoma cell lines investigated (WM115, WM266-4, NZM6, NZM9, NZM40). In the remainder of the melanoma cell lines this effect was not observed, although in some cases the melanoma cell lines did have a relatively high initial level of *EBF3*. Moreover, in the cell lines where the knockdown led to a reduction in migration or invasion, *EBF3* knockdown was also associated with reduced MTT values (WM115, WM266-4, NZM6, NZM9, NZM40, refer to Figure 5). We note that, as described previously for melanoma cell lines [25], the invasive and proliferative potentials of melanoma cell lines did not necessarily correlate with metastasis; several metastatic melanoma cell lines in this study were non-invasive. Therefore, despite our observation that *EBF3* expression levels in the cell lines were inconsistently correlated with the proliferative or invasive properties of the melanoma cell lines, it is worth noting the identified DNA methylation changes were metastasis-related, and not necessarily invasiveness-related. We propose that the increased *EBF3* methylation was associated with the aggressive phenotypic behavior in the metastatic melanoma cell lines, and it may be associated with an as yet unknown driver role of *EBF3* expression in melanoma metastasis (summarized diagrammatically in Figure 7F), rather than being involved in proliferation, migration, or invasion.

Known transcription factor binding sites near the differentially methylated *EBF3* promoter fragment include the aristaless-related homeobox protein (ARX), a transcriptional repressor, which is known to bind to the 5′ promoter region of *EBF3* at approximately 1,870 nucleotides upstream of the *EBF3* transcription start. The binding of ARX in neuronal cells was sufficient to repress endogenous expression of *EBF3* [38, 39]. Therefore ARX transcriptional repressor, or another factor that binds in this region, could be responsible for repressing *EBF3* expression. We hypothesize that transcriptional repression is abrogated by the *EBF3* promoter methylation, leading to elevated *EBF3* expression in metastatic melanoma.

This study demonstrates a possible approach to identify candidate epigenetic drivers. Additional studies involving greater numbers of paired primary/metastatic cancer samples will be required to identify further candidate epigenetic drivers of melanoma metastasis. From the present RRBS analysis of paired primary and metastatic melanoma cell lines, *EBF3* was identified as a candidate oncogenic metastasis epi-driver. A number of other common significant differential methylation changes between primary and metastatic melanomas were also identified, and will need further investigation to determine whether they may play a role in melanoma metastasis.

## MATERIALS AND METHODS

### Cell lines

Mel-ST cells (normal transformed melanocyte) were generously donated in January 2007 by Professor Robert Weinberg (MIT, USA), and cultured in DMEM plus 10% fetal bovine serum (FBS). This cell line has not since been tested for authentication. Four melanoma cell lines used in this study were obtained from America Type Culture Collection (Manassas, VA): WM115 (ATCC^®^ CRL-1675™), WM266-4 (ATCC^®^ CRL-1676^™^), Hs688(A).T (ATCC^®^ CRL-7425™), and Hs688(B).T ATCC^®^ CRL-7426™). These cells were received in October 2013, together with ATCC certificates of analysis and authentication. Low passage number cells were used at all stages of this project. These cells were cultured in Minimum Essential Medium (MEM-α) (Invitrogen) supplemented with 1% penicillin-streptomycin (Gibco, NY, USA) and 10% FBS. Four additional melanoma cell lines were generously provided by B. Baguley (University of Auckland) in May 2015; NZM6, NZM9 NZM11 and NZM40, and were cultured in MEM-α media supplemented with 1% penicillin-streptomycin, 5% FBS and 0.1% Insulin-transferrin-selenium (Roche). These cell lines have very recently been tested and authenticated. All cells were grown under standard conditions (5% CO_2_, 21% O_2_, 37°C, humidified atmosphere), except WM115, which were cultured at 35°C. One of the primary/metastasis cell line pairs (WM75 and WM373), which was included in our studies, was extremely slow growing. We received only DNA for these cell lines, which was generously provided to us by Patricia Brafford and Dr Meenhard Herlyn (The Wistar Institute, Philadelphia, USA). As a consequence, performing functional experiments with WM75 and WM373 was not feasible, and only DNA level data was reported for this cell line pair.

### Reduced representation bisulfite sequencing (RRBS)

RRBS libraries for the cell lines were prepared according to previously published methods [40–43]. Genomic DNA from cell pellets was extracted using a column-based purification kit and digested with *Msp*I enzyme. After end-repair and A-tailing, methylated adaptors were ligated to *Msp*I fragments. Adaptor-ligated fragments were size-selected (150–330 bp), bisulfite converted and PCR amplified (16–18 cycles). Following purification, libraries were quantified and the quality was assessed. Single-ended RRBS libraries with 100 bp read length were sequenced on an Illumina HiSeq2500.

### Processing and alignment of RRBS data

Sequenced reads were assessed using FastQC. Quality trimming and adaptor trimming were performed using the fastq_quality_trimmer and our in-house cleanadaptors program [44], respectively. The sequenced reads were mapped against the complete human reference genome GRCh37 using the Bismark v0.6.4 alignment tool [17] with a stringent criteria of one mismatch (default = 2) in the seed (i.e., in the first 28 bp of the sequenced reads) . The alignments were performed on a Macbook Pro with 64 bit duo quad core Intel Xeon processors and with 22 Gb RAM running MacOS 10.6.

### DNA methylation data analysis

Following alignment, fragment-based methylomes were generated using DMAP [19]. Correlation analysis of replicates and initial hierarchical clustering analysis was performed with methylKit [45]. Differential methylation fragment (DMF) analysis of the different groups was performed using DMAP [19] and statistical analysis used a Fisher's exact test with Bonferroni correction. Comparison of common driver DMFs with ENCODE data was performed using Epiexplorer [46] as previously described [47]. The Epiexplorer tool contains genome regulation data for nine cell lines (GM12878, H1hESC, HepG2, HMEC, HSMM, HUVEC, K562, NHEK and NHLF) and we used the combined features of all nine cell lines for the analysis described here. DAVID [48] was used for functional annotation analyses of DMF-associated genes.

### Sequenom experiments

Primers were designed using the EpiTYPER assay designer ( http://www.epidesigner.com/). The sequences of the primers are given in Supplementary Table S7 (in supplementary information file). One microgram genomic DNA was bisulfite converted using the EZ DNA methylation kit (Zymo Research) according to the “Alternative Cycle Protocol” as described by the manufacturer. PCR was performed with the following cycling protocol: 15 min at 94°C, 45 cycles of (20 s at 94°C, 30 s at optimal annealing temperature, 1 min at 72°C), 3 min at 72°C for extension and hold at 4°C. Mass spectrometry-based EpiTYPER-Sequenom assays were performed using the manufacturers’ protocol on one 384-well plate.

### TCGA DNA Methylation and RNA-Seq data analysis

For analysis of SKCM-TCGA datasets, normalized beta methylation values and normalized RNA-Seq read counts for *EBF3* were extracted. The datasets were segregated into primary and metastatic tumors for comparative analysis. The Shapiro-Wilk test was performed to assess the normal distribution of methylation data and a *t*-test was then performed. For the majority of sites the data were not distributed normally, and therefore a Mann-Whitney *U* test was also performed. These analyses were performed using the scan_tcga set of tools [49].

### Gene knockdowns

siRNA-mediated knockdown of *EBF3* was performed by using reverse transfection with Lipofectamine RNAiMAX (Invitrogen) as previously described [25]. ON-TARGETplus SMARTpool *EBF3* siRNA (siEBF3) (Dharmacon Research, Lafayette, CO) was used to knockdown *EBF3* mRNA levels. Sequences were 5′-GCAGGCAACCCUCGAGAUA-3′. 5′-CAUCAUAAUUGGCGACAAC-3′, 5′-UCUUUGAUCUGUUUCGUUA-3′, 5′-CAACCAUAGAUUACGGCUU-3′. The ON-TARGET-plus Nontargeting Pool (siNeg) (Dharmacon Research, Lafayette, CO) was used as a negative control. 2 μl of Lipofectamine RNAiMAX and 2–10 nmol/L of siEBF3 were used for each well of a 6-well-plate, in 500 μl of OPTI MEM I media and 1500 μl of cells (3 × 10^5^/mL) suspended in the appropriate culturing medium, without the addition of antibiotics (penicillin-streptomycin), as this can interfere with the reverse transfection. Cells were transfected for 48 hours. Real-time PCR was used to determine the knockdown efficiency at 48 h, 72 h and 96 h post-transfection (in Hs688(A).T and WM115 cell lines). For WM115 the knockdown was maintained until after 96 h. For Hs688(A).T the knockdown was maintained until after 72 hours, but was lost at 96 hours post-transfection. Efficient knockdown was observed at 48 h and 72 h, but not 96 h post-transfection. Cell viability following *EBF3* knockdown was assessed by trypan blue exclusion assays, as described previously [50]. The mean cell viability for the *siEBF3* treated cells ranged from 86.5% to 95.2%, while the mean viability for siControl (siNeg) treated cells ranged from 88.5% to 95%.

### *EBF3* mRNA expression and gene expression associated with epithelial-mesenchyme transition (EMT)

Expression levels of mRNAs following knockdown were determined by (RT-QPCR), as previously described [25] using SYBR Green on an ABI 7300 Real-Time PCR System. Relative *EBF3* expression levels were analysed using the qBase software and the delta-delta-Cq method [51]. qPCR primers for *EBF3* expression were: F: 5′- AGATCTTGCTCTGTTCTGACTC-3′, and R: 5′- GCTTTTGTGGACTTTGTGGAG- 3′. Expression of *EBF3* was normalised to house keeping genes *RPL27* and *SPR14*. Primers for *RPL27* were: F: 5′- TGGCTGGAATTGACCGCTA- 3′, and R: 5′-CCTTGTGGGCATTAGGTGATTG-3′ and for SRP14 were: F: 5′- ACGGAGCTGACCAGACTTTTC -3′ and R: 5′- TGGTTCGACCGTCATACTTCTT -3′. Details of primers used for real time analysis of the six EMT related genes are given in Supplementary Table S16 [52].

### DNA methylation inhibition experiment on *EBF3*

Demethylation experiments: Demethylation in the described cell lines was induced with 5-Aza-2′-deoxycytidine (A3656, Sigma- Aldrich, St Louis, MO, USA). First, we performed a viability assay (based on trypan blue staining) at different concentrations of the drug and chose 5 μM as the optimal concentration to induce demethylation. Cells were treated at 5 μM for 72 h and DNA and RNA were extracted following this treatment. The experiments were performed in triplicate. RT-PCR experiments were performed for the *EBF3* gene as described previously [54].

Primers for bisulfite genomic sequencing were designed using the MethPrimer program [53] to include common differentially methylated fragments (DMFs) of the *EBF3* promoter (993 bp from the TSS); EBF_993_F: 5’-GGAGTTAATTGTTTTAAAAATTAA G- 3′ and EBF _993_R: 5′-ATTTTCTCTTAACAAAAAAATACCTAAAAC-3′. Amplified bisulfite genomic DNA products were cloned and individual cloned plasmids were sequenced using Sanger sequencing to confirm demethylation of the *EBF3* promoter, as described previously [18].

### MTT assays

MTT assays following knockdown were performed as previously described [54], except that 7 × 10^4^ cells were seeded in 96 well plates, and each assay was performed with at least 5 replicates. The MTT assay was normalized by cell counting, where the same number of cells were used for both *EBF3* knockdown and negative control assays.

### Invasion assays

Boyden chamber invasion assays were carried out as previously described [25], with the following modifications; 2.5 × 10^4^ cells were seeded into transwell inserts with 8μm micropore filters either without or with matrigel coating. Media containing 10% FBS was added to the lower chamber as the chemoattractant. Five random fields of view were captured per transwell insert to count the number of migrating cells.

## SUPPLEMENTARY MATERIALS FIGURES AND TABLES






