# Supplementary material for: Genome-wide methylation sequencing of paired primary and metastatic cell lines identifies common DNA methylation changes and a role for EBF3 as a candidate epigenetic driver of melanoma metastasis
Source: Oncotarget. 2016 Dec 20;8(4):6085–101. doi: 10.18632/oncotarget.14042 (PMC5351615; doi:10.18632/oncotarget.14042)
Supplement: Supplementary file 1 [file oncotarget-08-6085-s001.pdf]

Genome-wide methylation sequencing of paired primary and metastatic cell lines identifies common DNA methylation changes and a role for *EBF3* as a candidate epigenetic driver of melanoma metastasis

Supplementary Materials

SUPPLEMENTARY DATA

Supplementary Data Files S1 and S2: These data files provide detailed information on commonly deregulated hyper (Supplementary Data File S1) and hypomethylated (Supplementary Data File S2) fragments. The spreadsheets contain data for chromosome, length of the fragment, number of CpG sites contained within the fragment, methylation value of the cell lines in the fragment (0 = 0% methylation, 1 = 100% methylation), raw *P*-values for each paired comparison (Fisher's exact test), distance in relation to the gene (which are calculated from the start of the gene relative to the fragment, therefore negative values mean the DMF is inside the gene body and the value indicates how far downstream the DMF is from the TSS, while positive values indicate the distance of the upstream DMF to the nearest TSS), relationship of the DMFs with the gene (e.g., upstream, exon, intron) and the name of the nearest associated gene.

Supplementary Data Files S1: Description: Excel spread sheet containing information on 65 commonly hypomethylated DMFs in all metastatic cell lines compared to their matched primary cell lines. The spreadsheet provides data of chromosome, length of the DMFs, number of CpG sites contained within the DMF, methylation value of the cell lines in the fragment (0 = 0% methylation, 1 = 100% methylation), raw P-values for each paired comparison (Fisher's exact test), distance in relation to the gene (calculated from the start of the gene relative to the fragment. therefore negative value means the DMF is inside the gene body and the value indicates how far downstream the DMF is from the TSS, while positive value indicates the distance of the upstream DMFs to its nearest TSS), relationship of the DMFs with the gene (e.g., upstream, exon, intron) and the name of the associated gene. See Supplementary_Data_File_S1

Supplementary Data Files S2: Description: Excel spread sheet containing information on 10 commonly hypermethylated DMFs in all metastatic cell lines compared to their matched primary cell lines. The spreadsheet provides data of chromosome, length of the DMFs, number of CpG sites contained within the DMF, methylation value of the cell lines in the fragment (0 = 0% methylation, 1 = 100% methylation), raw P-values for each paired comparison (Fisher's exact test) , distance in relation to the gene (calculated from the start of the gene relative to the fragment. therefore negative value means the DMF is inside the gene body and the value indicates how far downstream the DMF is from the TSS, while positive value indicates the distance of the upstream DMFs to its nearest TSS), relationship of the DMFs with the gene (e.g., upstream, exon, intron) and the name of the associated gene. See Supplementary_Data_File_S2

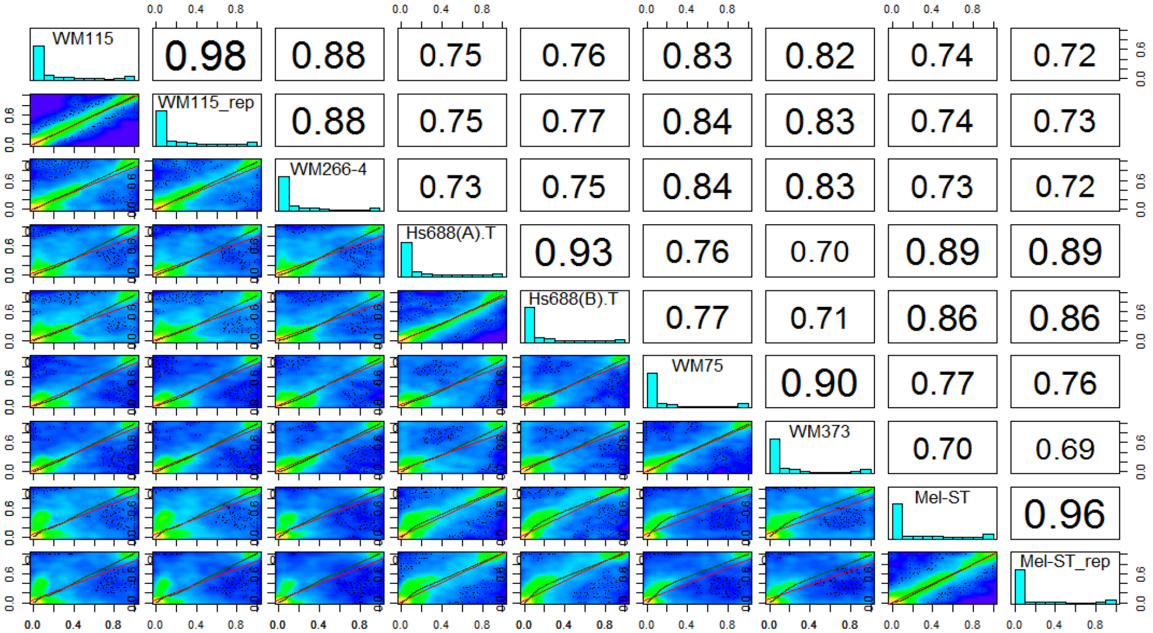

Supplementary Figure S1: CpG methylation Correlation Scatter plots of the analysed cell lines. Left is the scatter plot of CpG methylation values for each sample. The numbers on right denote pair-wise CpG base Pearson's correlation score of analysed samples. The histograms show the frequency of CpG site methylation. The X and Y axis scales of the plots were modified to 0.0 (complete unmethylation) to 1.0 (complete methylation) by methylKit, instead of 0–100 percent methylation.

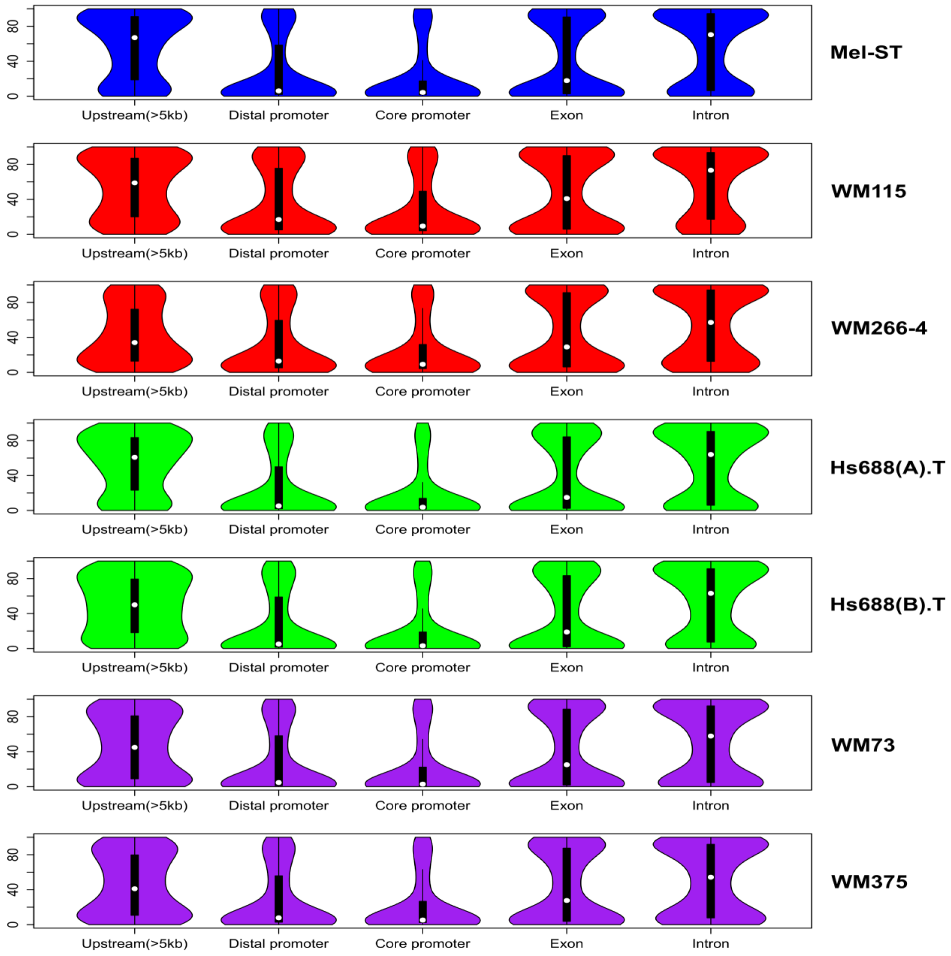

Supplementary Figure S2: DNA methylation distribution in different genomic elements in melanoma cells. These analyses were performed on the MspI fragments with high coverage as described above. Mel-ST (blue), WM115 and WM266-4 (in red, top and bottom respectively), Hs688(A).T and Hs688(B).T (in green, top and bottom respectively), WM73 and WM375 (in purple, top and bottom respectively). Two cell line pairs (Hs688(A).T, Hs688(B).T and WM75 and WM73) exhibited similar methylation profiles across all genomic elements (See also Supplementary Table S4). However, WM266-4 showed loss of both gene body and intergenic methylation (> 5 kb upstream) compared to its matched primary cell line (WM115). Further, WM115 and WM266-4 cells showed notable hypermethylation in core (0–2 kb upstream from the start of the gene) and distal promoters (0–5 kb upstream from the start of the gene) and in exons, compared to the other two cell line pairs, or Mel-ST cells. (Note, quantification of methylation for each element is provided in Supplementary Table S4).

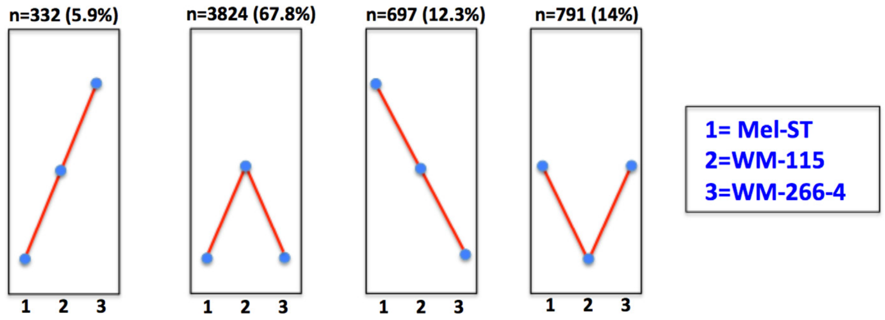

Supplementary Figure S3: Schematic of changes in methylation patterns in the common differentially methylated fragments (DMFs), as exemplified by Mel-ST vs WM115 and WM115 vs WM266-4. A total of 5644 fragments were differentially methylated between these cell line comparisons (DMFs). The directions of the methylation changes in the comparisons are shown here. The changes are not drawn to scale.

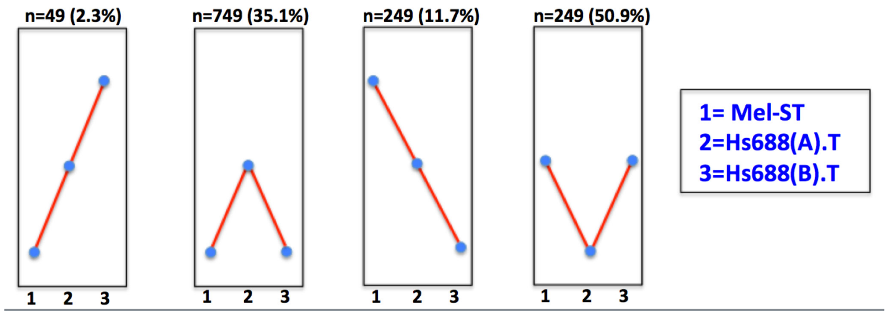

Supplementary Figure S4: Schematic of changes in methylation patterns in the common differentially methylated fragments (DMFs), as exemplified by Mel-ST vs Hs688(A).T and Hs688(A).T vs Hs688(B).T. A total of 2133 fragments were differentially methylated between these cell line comparisons (DMFs). The directions of the methylation changes in the comparisons are shown here. The changes are not drawn to scale.

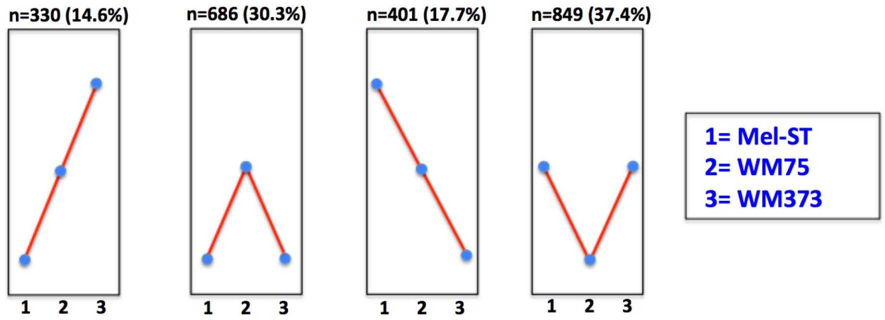

Supplementary Figure S5: Schematic of changes in methylation patterns in the common differentially methylated fragments (DMFs), as exemplified by Mel-ST vs WM75 and WM75 vs WM373. A total of 2266 fragments were differentially methylated between these cell line comparisons (DMFs). The directions of the methylation changes in the comparisons are shown here. The changes are not drawn to scale.

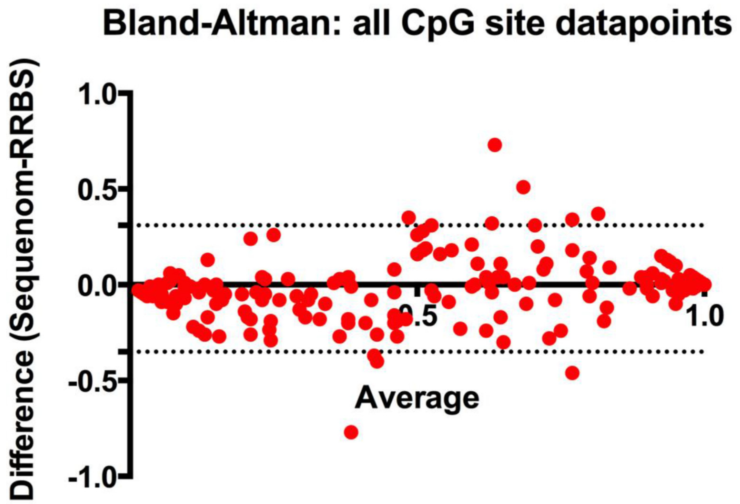

Supplementary Figure S6: Bland–Altman plot (mean difference plot) showing the limits of agreement between the two methods (RRBS and Sequenom) at single CpG level. Each red dot represents a CpG site. The solid black line shows the mean methylation values of the replicates. The dotted black lines (top and bottom) represent the 95% confidence intervals (mean ± 1.96 SD).

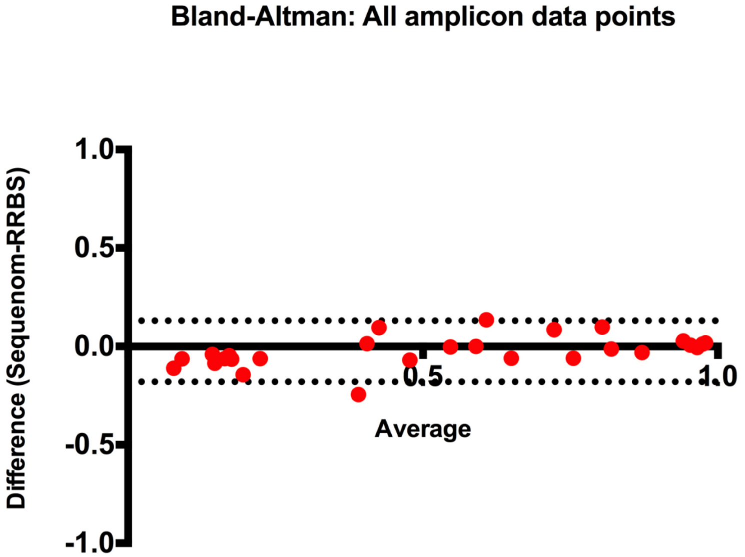

Supplementary Figure S7: Bland–Altman plot (mean difference plot) showing the limits of agreement between the two methods (RRBS and Sequenom) over the common amplicon. Each red dot represents a CpG site. The solid black line shows the mean methylation values of the replicates. The dotted black lines (top and bottom) represent the 95% confidence intervals (mean ± 1.96 SD).

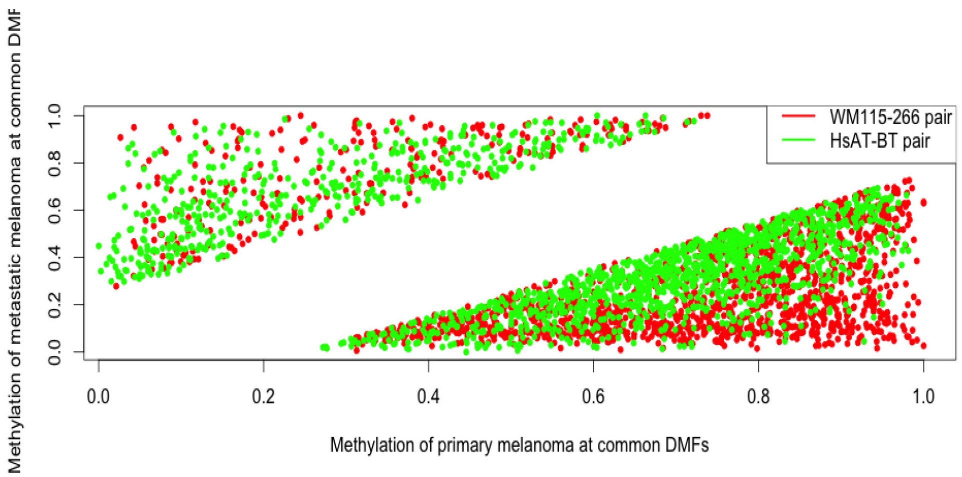

Supplementary Figure S8: X-Y methylation plot of the common DMFs between WM115 vs WM266-4 (red) and Hs688(A).T vs Hs688(B).T. (green) pair comparisons.

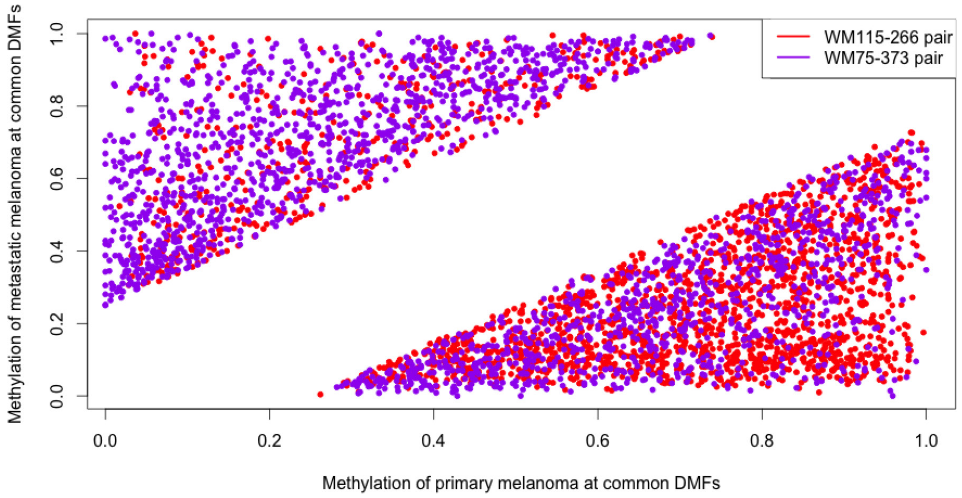

Supplementary Figure S9: X-Y methylation plot of the common DMFs between WM115 vs WM266-4 (red) and WM75 vs WM373 pair comparisons (purple).

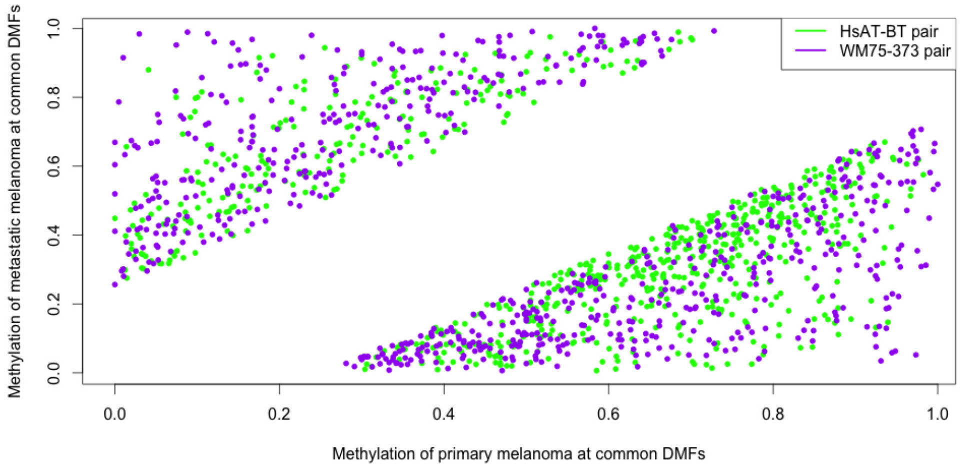

Supplementary Figure S10: X-Y methylation plot of the common DMFs between Hs688(A).T vs Hs688(B).T (green) and WM75 vs WM373 pair comparisons (purple).

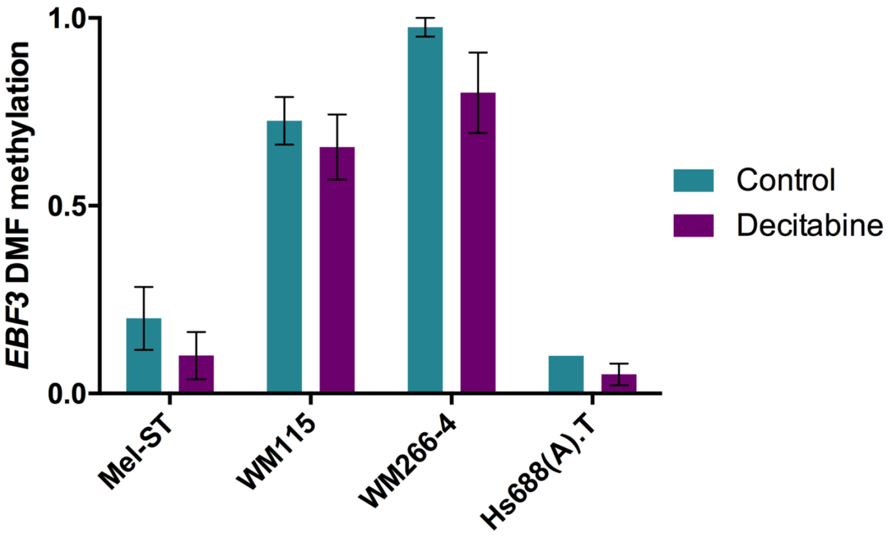

Supplementary Figure S11: Analysis of DMF methylation levels in the *EBF3* promoter (-993 bp) before and after decitabine treatment (at 72 h). The average methylation levels in the *EBF3* promoter DMF (at -993 bp) in multiple clones derived from four different cell lines (Mel-ST, WM115, WM266-4 and Hs688(A).T) were detected using locus-specific bisulfite sequencing at 72 h following decitabline treatment. In all cases decitabine treatment reduced the average methylation level in the *EBF3* promoter DMF. For Hs688(A).T, one bisulfite clone was obtained for the control treatment, therefore error bars were not included for this cell line treatment. The error bars represent mean ± standard error of mean.

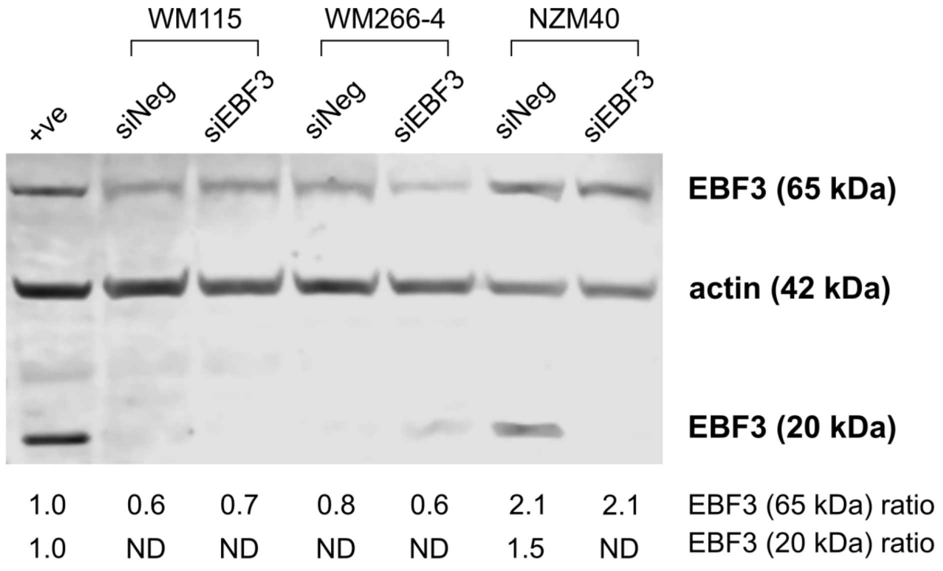

Supplementary Figure S12: Representative Western blot of three melanoma cell lines before and after *EBF3* knockdown. After 48 hours in the presence of siEBF3 or siNEG, protein was extracted from cell pellets and stored at −80°C in the presence of protease inhibitors. In each lane of a precast 4–12% SDS PAGE gel, 80 µg of total protein was loaded. Following gel electrophoresis, the separated proteins were transferred to nitrocellulose membrane using the iBlot transfer system and blocked with milk solution. The membrane was incubated overnight in mouse anti-EBF3 and rabbit anti-actin primary antibody solutions. Following membrane washing, the membrane was incubated with anti-mouse and anti-rabbit infrared dye secondary antibodies. The membrane was imaged and bands quantified using two-colour detection on an Odyssey imaging system. A 65KDa EBF3 band was detected in all samples and an additional 20KDa band was observed in the NZM40 cell line. The Western blot shows knockdown of the 65 kDa EBF3 in the WM266-4 cell line and complete ablation of the 20 kDa EBF3 in the NZM40 cell line.

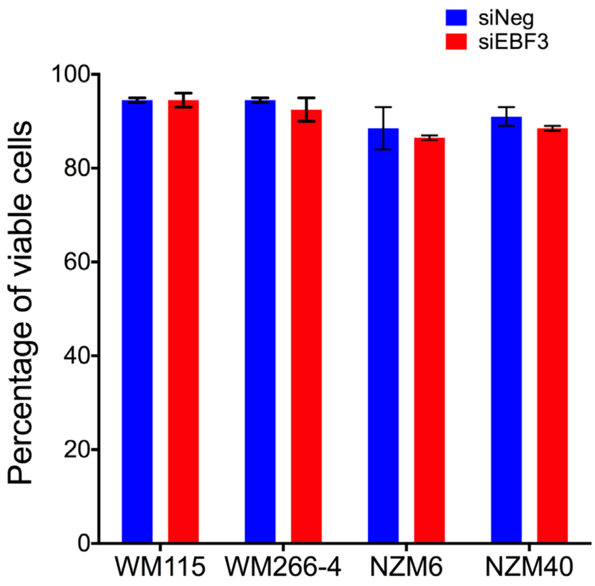

Supplementary Figure S13: Cell viability assay comparison between negative control (siNeg) and *EBF3* knockdown (siEBF3). The percentage of viable cells upon *EBF3* knockdown was assessed at 72 hours following siRNA transfection. Data for WM115, WM266-4, NZM6 and NZM40 are presented here as representative examples; these cell lines had shown a reduction in MTT OD (560 nm) following *EBF3* knockdown (see Figure 6).

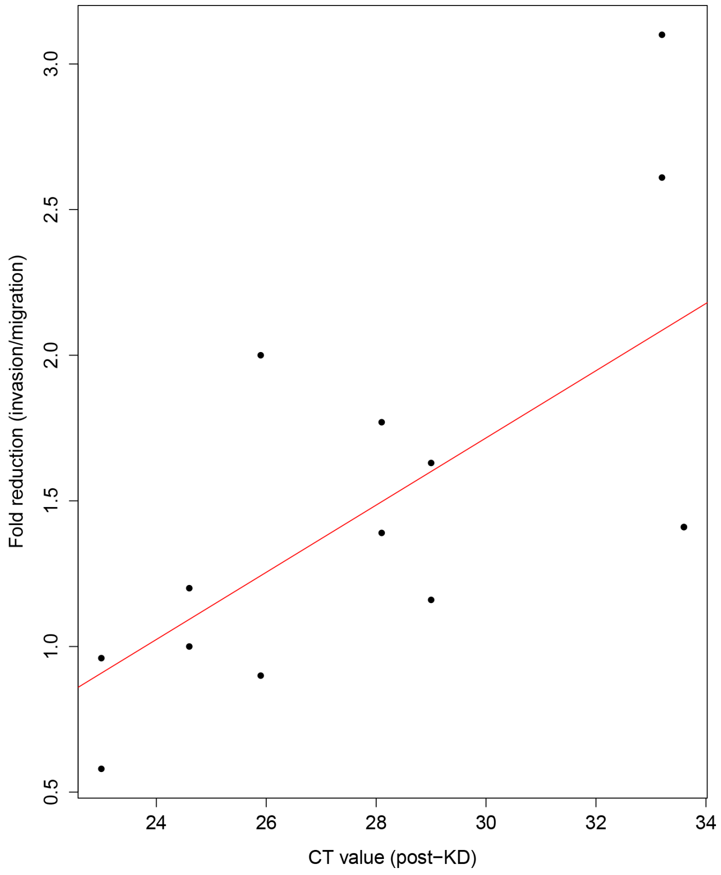

Supplementary Figure S14: Relationship between *EBF3* expression levels and change in cell migration or invasion in the analysed cell lines. Y-axis: fold change in migration and invasion after *EBF3* knockdown. X-axis: CT value after *EBF3* knockdown as determined by qPCR.

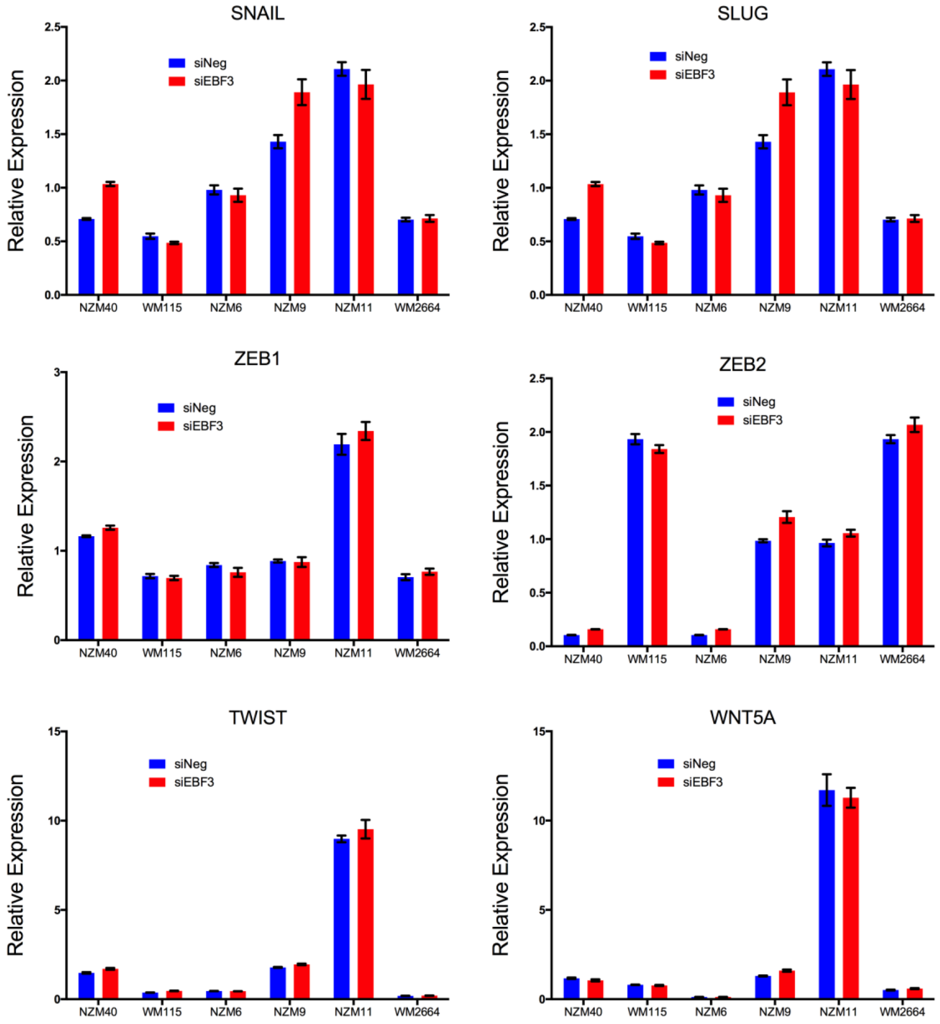

Supplementary Figure S15: Relative expression of key EMT marker genes following transfection with negative control (siNeg) and *EBF3* knockdown (siEBF3) siRNAs. Six EMT markers including *SNAIL*, *SLUG*, *ZEB1*, *ZEB2*, *TWIST* and *WNT5A* were investigated upon knockdown of *EBF3*.

Supplementary Table S1: Details of the cell lines included in the study

Cell lines	Cancer phenotype	Source	Date of acquisition
Mel-ST	Normal transformed melanocyte	Prof. Robert Weinberg	Jan-2007
WM115	Primary	ATCC	Oct - 2013
WM266-4	Metastatic	ATCC	Oct - 2013
Hs688(A).T	Primary	ATCC	Oct - 2013
Hs688(B)T	Metastatic	ATCC	Oct - 2013
WM75	Primary	DNA received	N/A
WM373	Metastatic	DNA received	N/A
NZM6	Metastatic	Prof B Baguley	May - 2015
NZM9	Metastatic	Prof B Baguley	May - 2015
NZM40	Metastatic	Prof B Baguley	May - 2015
NZM11	Metastatic	Prof B Baguley	May - 2015

Supplementary Table S2: Summary of RRBS alignment for melanoma cell lines[#]

Cell lines	Sequenced reads	Reads after QC check and adaptor cleaning	Uniquely mapped reads (%)	Multiple mapping (%)
Mel-ST1	12,815,958	12189734	7730017 (63.4%)	16.3
Mel-ST2	5,011,128	3660824	2328467 (63.6%)	15.7
WM115	18650627	18248660	12345000 (67.6%)	18.1
WM266-4	26720918	25322840	16777256 (66.3 %)	18.1
Hs688(A).T	22829285	21819053	14293849 (65.5 %)	17.8
Hs688(B).T	24207118	23180151	15074280 (65.0 %)	18.7
WM115_2	25912073	25550928	17153693 (67.1 %)	19.3
WM75	14,999,356	14940053	8067321 (54%)	15.2
WM373	21375898	20575750	13936497 (67.7%)	18.9

[#]After trimming the 3 filled-in bases from the 3’ ends of the reads. The reads were mapped against the complete human genome GRCh37. The mapping runs were performed on a Mac Pro with 64 bit duo quad core Intel Xeon processors and with 22Gb RAM running MacOS 10.6 and later MacOS 10.7. *Reads after QC check and adaptor cleaning.

Supplementary Table S3: Global DNA methylomes of melanoma cell lines (RRBS)

Cell lines	Number of analysed MspI fragments with high coverage[#]	Number of CpG sites in analysed fragments	Mean methylation of the MspI fragments
Mel-ST	279074	1492416	50.96
WM115	427567	2190041	53.26
WM266-4	365305	1878526	46.04
Hs688(A).T	307090	1568858	48.43
Hs688(B).T	370148	1893552	47.29
WM75	248578	1547355	45.21
WM373	329042	2108766	45.13

[#]These fragments contained 10 or more sequenced reads in at least 2 CpG sit.

Supplementary Table S4: Methylation of major genomic elements in the melanoma cells

Cell line	Upstream (> 5kb)		Distal promoter		Core promoter		Exon		Intron	
	Mean	Median	Mean	Median	Mean	Median	Mean	Median	Mean	Median
Mel-ST	57.02	67.05	28.13	6.08	20.11	4.33	42.21	17.86	55.23	70.42
WM-115	54.12	58.86	36.74	16.87	28.25	9.40	46.71	40.82	58.88	73.27
WM_266	42.59	34.09	31.74	12.9	24.72	9.20	44.60	29.06	53.87	57.14
Hs688(A).T	53.92	60.81	25.6	5.19	17.70	3.70	39.48	14.90	52.86	64.00
Hs688(B).T	49.03	50.0	27.73	5.14	19.42	3.21	39.45	18.88	53.44	63.16
WM75	45.36	41.1	28.65	7.86	21.55	5.26	42.50	27.66	50.92	54.29
WM373	46.06	44.9	27.64	4.65	19.99	2.80	41.66	25.00	51.05	57.83

Supplementary Table S5: Summary of differential methylation analysis between melanoma cells#

Comparison	Analysed Fragments	Adjusted *P*-cutoff [1]	Fragment+ 25%diff [2]	% DMFs among analysed	Number of Hyper DMFs[3]	% Hyper[3]
Mel-ST_vs_ WM115comb	170898	5.85144E-08	23417	14%	12387	53% (Mel-ST)[4]
WM115comb_vs_266	215269	4.64535E-08	22745	11%	19243	85% (WM115)
Mel-ST_vs_Hs688(A).T	160111	6.24567E-08	9527	6%	7065	74% (Mel-ST)[4]
Hs688(A).T_vs_BT	178537	5.60108E-08	7220	4%	4887	68% (Hs688(A).T)
Mel-ST_vs_WM75	139136	7.18721E-08	17341	12%	10751	62% (Mel-ST)[4]
WM75_vs_WM373	146728	6.81533E-08	7520	5%	3670	49% (WM75)

[1]*P* value: The significance level was set at 0.01. Bonferonni correction was applied for multiple test correction. Adjusted *P*-value = 0.01/number of analysed fragments. [2]Fragments that passed the P-value cut off and also showed a mean methylation difference of 25% or higher on the entire fragment. [3]Percentage of DMFs that showed hypermethylation, the name of the sample in the brackets is the sample that showed hypermethylation in the DMFs. [4]These analyses suggested that Mel-ST has a higher number of hypermethylated DMFs compared to the primary tumour cell lines. In other words, primary melanoma cell lines were relatively hypomethylated compared to normal melanocyte cell line.

Supplementary Table S6: Comparison of sequenom and RRBS assays

Nearest Gene	Feature	Expression data (public)	Location: RRBS	Location Sequenom	No. CpGs: RRBS	No. CpGs: Sequenom	No CpGs overlapping between assays
CBX8	Promoter	yes (Microarray, SAGE)	chr17: 77776861- 77777095	chr17: 77776756-77777210	15	26	14
HES5	Promoter, CGI core	yes (Microarray, SAGE)	chr1: 2464123-2464460	chr1: 2464112-2464432	13	12	12
EXOC3L2	Promoter	yes (Microarray, SAGE)	chr19: 45737587- 45737939	chr19: 45737388- 45737777	26	51	13
POU3F2	Promoter, CGI core	yes (Microarray)	chr6:99,279,554-99,279,698	chr6:99,279,628-99,279,566	19	18	13
Total					73	107	52

Supplementary Table S7: Details of Sequenom primers used for validation experiments with 5 genes

Gene	Sequence Description	Primer Sequence
CBX8	F	aggaagagagGTTGGTGTTGTTTGTGAGATTTA
	T7R	cagtaatacgactcactatagggagaaggctAAATCTAAAATCAAACTCTCCACTC
HES5	F	aggaagagagGGGAGATGTGGTTTAATTTTTTTAT
	T7R	cagtaatacgactcactatagggagaaggctCACACTTTAACCCCATTCACAATA
EXOC3L2	F	aggaagagagAGTTAGTGTTAGGGGGTAGGGTTGA
	T7R	cagtaatacgactcactatagggagaaggctCAACTAAACCAAATACTAATACAAAAAAAA
POU3F2	F	aggaagagagTGGGAGGTAGATAAGGAAGTTTAGG
	T7R	cagtaatacgactcactatagggagaaggctAAAATACACAAAAAAATAACCTACCC

Supplementary Table S8: Common differentially methylated fragments (DMFs) between paired primary and metastatic melanoma cell lines

Primary-metastatic pair comparisons	Common DMFs	Overlap % of the common DMFs
WM115-WM266 & Hs688(A).T-Hs688(B).T	1644	22.70
WM115-WM266 & WM75-WM373	2123	28.23
Hs688(A).T-Hs688(B).T & WM75-WM373	734	10.2

Supplementary Table S9: Details of the common DMFs between paired primary and metastatic melanoma cell lines

Primary-metastatic pair comparisons	Common DMFs	Hypermethylated in both metastatic samples	Hypomethylated in both metastatic samples	Methylation change in same direction in both metastatic samples#
WM115-WM266 & Hs688(A).T-Hs688(B).T	1644	115	1061	72%
WM115-WM266 & WM75-WM373	2123	230	766	47%
Hs688(A).T-Hs688(B).T & WM75-WM373	734	116	325	60.1%

#These fragments were either hypomethylated or hypermethylated in both primary melanoma cell lines compared to the matched metastatic cell lines.

Supplementary Table S10: Comparison of chromatin maps and genome regulation features of hyper-and hypomethylated common driver DMFs[#]

Genome Feature	Proportion of overlapping hypermethylated common DMFs	Proportion of overlapping hypomethylated common DMFs
Active promoters	0.4	0.15
Poised promoters	0.2	0.14
DNaseI sites	0.8	0.19
H2AZ	0.8	0.66
H3K27ac	1	0.46
H3K27me3	0.8	0.85
H3K36me3	0.9	0.43
H3K4me1	1	0.54
H3K4me2	0.9	0.49
H3K4me3	0.9	0.42
H3K79me2	0.8	0.2
H3K9ac	1	0.48
H3K9me	0.7	0.46
H3K9me3	0.3	0.74
H4K20me1	1	0.75
Heterochromatin	0.4	0.91
Strong enhancers	0.4	0.19
Transcriptional transition	0.2	0.02
Weak enhancers	0.5	0.17
Weak transcribed	0.3	0.49
Repeat elements	0	0.48
Conserved regions	0.3	0.09

[#]The overlap of hyper- and hypomethylated fragments with a genome feature (using publicly available data) is calculated on a scale of 0 to 1. This comparison was also made against aggregated data from all nine cell lines available in the ENCODE project (as the melanocyte cell line was not available to make a direct comparison) and this is referred to as “any tissue” analysis in the Epiexplorer platform.

Supplementary Table S11: Comparison of transcription factor binding sites present in hyper- and hypomethylated common driver DMFs[#]

Transcription factor binding sites	Percent of overlapping hypermethylated common DMFs	Percent of overlapping hypomethylated common DMFs
CTCF	50%	43.10%
EGR1	10%	7.70%
GABP	40%	6.20%
Pol2	50%	15.40%
Pol2b	70%	40%
SIN3AK20	20%	3.10%
SP1	10%	6.20%
TAF1	30%	4.60%

Supplementary Table S12: Gene ontology enrichment of commonly hypermethylated genes in metastatic melanoma[1]

Annotation term	Number of genes	Genes	*P* value[2]
**Biological Process**			
Cytoskeleton organization	2	*SIPA1, CNN2*	0.1785
Intracellular signaling cascade	2	*SIPA1, PMEPA1*	0.4428
Regulation of transcription	2	*EBF3, PDCD6*	0.5696
**Molecular Function**			
Transcription regulator activity	2	*EBF3, PDCD6*	0.3906
Metal ion binding	2	*EBF3, PDCD6*	0.7848
**Cellular Compartment**			
Extrinsic to membrane	2	*SIPA1, PDCD6*	0.1789
Endoplasmic reticulum	2	*PDCD6, TECR*	0.3232
Integral to membrane	3	*SPNS2, TECR, PMEPA1*	0.6875
**Protein Domain**			
bHLH dimerisation region	2	*EBF3, PDCD6*	0.0338

[1]DAVID was used for gene ontology enrichment of 8 unique gene IDs.
[2]DAVID uses a modified Fisher's exact test for enrichment evaluation analysis.

Supplementary Table S13: Gene ontology enrichment of commonly hypomethylated genes in metastatic melanoma[1]

Annotation term	Number of genes	Genes	*P* value[2]
**Biological Process**			
Regulation of cell size	4	*FGFR2, TNN, NRG1, ENO1*	0.0033
Positive regulation of cell differentiation	4	*APOB, ROBO2, NRG1, HOXD11*	0.0044
Cell motion	5	*APOB, CCR6, DAB1, ROBO2, TNN*	0.0046
Cell adhesion	5	*EGFR, DAB1, NRXN2, ROBO2, TNN*	0.0177
Positive regulation of cell proliferation	4	*EGFR, FGFR2, NRG1, VSX2*	0.0220
Regulation of transcription	9	*CCR6, EFCAB6, ESRRG, NRG1, VSX2, HMGA1, KCNIP3, HOXD11, ENO1*	0.0257
Cellular macromolecular complex disassembly	2	*NRG1, HMGA1*	0.0378
Positive regulation of protein kinase activity	3	*EGFR, DAB1, NRG1*	0.0423
**Molecular Function**			
Transcription regulator activity	7	*ESRRG, NRG1, VSX2, HMGA1, KCNIP3, HOXD11, ENO1*	0.0175
Protein heterodimerization activity	3	*EGFR, APOB, ROBO2*	0.0365
Calcium ion binding	5	*EFHC2, PITPNM2, EFCAB6, NRXN2, KCNIP3*	0.0411
**Cellular Compartment**			
Clathrin-coated endocytic vesicle membrane	2	*EGFR, APOB*	0.0106
Extracellular space	5	*EGFR, WNT5A, APOB, TNN, NRG1*	0.0111
Cytosol	6	*ARHGEF4, TCP10, GYG2, HMGA1, KCNIP3, ENO1*	0.0258
**Protein Domain**			
Immunoglobulin subtype 2	3	*FGFR2, ROBO2, NRG1*	0.0376
EF-Hand type	3	*EFHC2, EFCAB6, KCNIP3*	0.0484
**KEGG Pathway**			
Regulation of actin cytoskeleton	4	*EGFR, ARHGEF4, FGFR2, FGF14*	0.0072
Pathways in cancer	4	*EGFR, WNT5A, FGFR2, FGF14*	0.0227

[1]DAVID was used for gene ontology enrichment of 28 unique gene IDs.

[2]DAVID uses a modified Fisher's exact test for enrichment evaluation analysis.

Supplementary Table S14: Extended methylation analysis of commonly hypomethylated DMFs with SKCM TCGA data for 458 patients

#chr	Start	End	Gene	Mean methylation (primary)	Mean methylation (metastatic)	Mann-Whitney U (*P*-Value)	*Welch's -t* (*P*-Value)
12	123469208	123469309	*PITPNM2*	0.39	0.38	0.8044	0.6241
22	44136692	44136821	*EFCAB6***	0.58	0.52	0.0055	0.0059
11	64428398	64428457	*NRXN2*	0.71	0.70	0.8144	0.3555
1	217263773	217263881	*ESRRG**	0.60	0.55	0.0421	0.0444
19	7680324	7680379	*CAMSAP3*	0.66	0.63	0.2330	0.2051
14	74725236	74725346	*VSX2*	0.19	0.19	0.2100	0.3745
10	102778620	102778689	*PDZD7*	0.26	0.28	0.2060	0.1732
6	167789062	167789194	*TCP10*	0.55	0.55	0.8960	0.8264
3	55517224	55517342	*WNT5A*	0.57	0.56	0.4654	0.4407
6	167530266	167530355	*CCR6*	0.70	0.67	0.3527	0.2128
10	123353267	123353349	*FGFR2*	0.66	0.66	0.6817	0.8470
7	55089734	55089778	*EGFR*	0.85	0.81	0.4062	0.0457
1	8936678	8936780	*ENO1*	0.73	0.69	0.0529	0.0556
2	176971324	176971421	*HOXD11*	0.32	0.29	0.1297	0.1792
15	93017078	93017209	*C15orf32*	0.58	0.59	0.9339	0.5974
X	2748125	2748180	*GYG2****	0.33	0.41	0.0001	0.0000
3	111718457	111718564	*TAGLN3*	0.45	0.48	0.3371	0.3402
2	21266953	21267028	*APOB*	0.56	0.56	0.9062	0.9245
6	34203665	34203774	*HMGA1*	0.29	0.31	0.9890	0.6052
7	1756030	1756166	*AC0743896***	0.52	0.45	0.0012	0.0015
7	985719	985798	*CYP2W1*	0.78	0.79	0.5271	0.6810

**P*-value < 0.05, ***P*-value < 0.01, ****P*-value < 0.001.

Supplementary Table S15: Extended methylation analysis of commonly hypermethylated DMFs with SKCM TCGA data for 458 patients

#chr	Start	End	Gene	Mean methylation (primary)	Mean methylation (metastatic)	Mann-Whitney U (*P*-Value)	*Welch's -t* (*P*-Value)
10	131763530	131763587	*EBF3***	0.09	0.11	0.0015	0.0031
11	65414680	65414818	*SIPA1****	0.66	0.73	0.0000	0.0003
19	14673048	14673096	*TECR**	0.30	0.34	0.0461	0.0709
20	56273715	56273790	*PMEPA1*	0.36	0.34	0.4755	0.4344
5	322864	322936	*PDCD6*	0.80	0.79	0.9756	0.8177

*P-value < 0.05, **P-value < 0.01, ***P-value < 0.001.

Supplementary Table S16: Details of the primers used for RT-PCR analysis for EMT related genes

Gene	Sequence (5′-3′)	Reference (as cited in the main article)
SNAIL	F1: CACTATGCCGCGCTCTTTC<br>R1: GGTCGTAGGGCTGCTGGAA	Xiong 2012
SLUG	F1: AAACTACAGCGAACTGGACACA<br>R2: GCCCCAAAGATGAGGAGTATC	Xiong 2012
TWIST1	F1: AGTCCGCAGTCTTACGAGGA<br>R1: GCCAGCTTGAGGGTCTGAAT	Xiong 2012
ZEB1	F2: GCCAATAAGCAAACGATTCTG<br>R1: TTTGGCTGGATCACTTTCAAG	Xiong 2012
ZEB2	F1: CGGTGCAAGAGGCGCAAACA<br>R1: GGAGGACTCATGGTTGGGCA	Xiong 2012
WNT5A	F1: GGGAGGTTGGCTTGAACATA<br>R2: AGGGCTCAGTGTGAAGAGGA	

F1: Forward primer.
R1: Reverse Primer.

REFERENCE

Xiong 2012. Roles of STAT3 and ZEB1 Proteins in E-cadherin Down-regulation and Human Colorectal Cancer Epithelial-Mesenchymal Transition http://www.ncbi.nlm.nih.gov/pubmed/22205702.
