# Supplementary material for: Genome-wide methylation sequencing of paired primary and metastatic cell lines identifies common DNA methylation changes and a role for EBF3 as a candidate epigenetic driver of melanoma metastasis
Source: Oncotarget. 2016 Dec 20;8(4):6085–101. doi: 10.18632/oncotarget.14042 (PMC5351615; doi:10.18632/oncotarget.14042)
Supplement: Supplementary file 2 [file oncotarget-08-6085-s002.pdf]

Description: Excel spread sheet containing information on 65 commonly hypomethylated DMFs in all metastatic cell lines compared to its matched primary cell lines. The spreadsheet provides data of chromosome, length of the DMFs, number of CpG sites contained within the DMF, methylation value of the cell lines in the fragment (0=0% methylation, 1=100% methylation), raw P-values for each paired comparison (Fisher's exact test) , distance in relation to the gene (calculated from the start of the gene relative to the fragment. therefore negative value means the DMF is inside the gene body and the value indicates how far downstream the DMF is from the TSS, while positive value indicates the distance of the upstream DMFs to its nearest TSS), relationship of the DMFs with the gene (e.g., upstream, exon, intron) and the name of the associated gene.

#Chr	Start	End	Length of the DMFs	Number of CpG sites in the DMFs	WM115	WM266-4	P-value	Hs688(A).T	Hs688(B).T	P-value	WM75	WM373	P-value	Distance from the start of the gene	Relation with gene	Gene name
3	77289501	77289557	57	9	0.8049	0.0939	3.66E-101	0.7534	0.3777	2.74E-22	0.9509	0.3275	1.04E-52	-1333710	on_intron	ROBO2
3	77215802	77215868	67	6	0.8386	0.3259	8.08E-40	0.736	0.3946	1.64E-09	0.9552	0.5514	1.51E-17	-1260021	on_intron	ROBO2
3	77161417	77161486	70	6	0.9201	0.4798	1.50E-45	0.7755	0.4231	1.30E-15	0.8824	0.5909	3.75E-08	-1205639	on_intron	ROBO2
1	57809361	57809439	79	10	0.7806	0.0601	5.22E-77	0.8414	0.5864	8.87E-12	0.3904	0.0303	1.81E-18	-1203044	on_intron	DAB1
3	77139597	77139663	67	7	0.9354	0.3636	5.82E-43	0.8065	0.4938	4.68E-10	0.9247	0.4129	8.99E-26	-1183816	on_intron	ROBO2
13	102473390	102473457	68	6	0.94	0.5765	1.95E-09	0.8455	0.4615	2.60E-10	0.49	0.1193	1.92E-11	-580733	on_intron	FGF14
8	31883320	31883385	66	6	0.8304	0.5583	1.36E-08	0.6803	0.375	3.48E-11	0.6615	0.0719	1.80E-18	-386482	on_intron	NRG1
7	51090083	51090181	99	8	0.795	0.0851	0	0.7115	0.1474	9.17E-52	0.88	0.2989	2.02E-35	-294431	on_intron	COBL
12	123469208	123469309	102	8	0.6299	0.3169	1.61E-18	0.5756	0.1286	1.49E-39	0.7321	0.4225	1.58E-13	-165353	on_exon	PITPNM2
X	44112178	44112259	82	5	0.9417	0.3398	5.83E-23	0.8068	0.1071	5.38E-25	0.8636	0.3438	4.42E-08	-90744	on_intron	EFHC2
22	44136692	44136821	130	5	0.8685	0.301	1.92E-46	0.7374	0.1835	4.56E-19	0.7899	0.2121	7.13E-21	-71524	on_intron	EFCAB6
11	64428398	64428457	60	6	0.9341	0.5678	3.38E-30	0.6297	0.0603	2.63E-56	0.8667	0.5303	8.90E-14	-62261	on_intron	NRXN2
1	217263773	217263881	109	7	0.9271	0.3942	9.70E-58	0.6715	0.4021	7.26E-12	0.9405	0.1888	5.47E-36	-47323	on_intron	ESRRG
1	175067537	175067605	69	5	0.7171	0.4599	3.13E-12	0.8071	0.5086	1.34E-14	0.7033	0.1215	1.23E-24	-30610	on_exon	TNN
2	95989732	95989810	79	6	0.8595	0.3131	7.04E-29	0.8269	0.44	2.02E-10	0.9733	0.6077	2.67E-10	-26757	on_intron	KCNIP3
19	7680324	7680379	56	6	0.5905	0.2452	5.89E-20	0.6577	0.3596	9.73E-12	0.3221	0.0711	6.24E-10	-19590	on_exon	CAMSAP3
14	74725236	74725346	111	9	0.7399	0.1296	7.43E-71	0.4212	0.0615	8.74E-20	0.8803	0.6207	7.15E-09	-19170	on_intron	VSX2
2	131609033	131609106	74	7	0.4899	0.2334	2.85E-15	0.6626	0.2227	6.36E-29	0.6467	0.3371	6.90E-10	-14616	on_intron	ARHGEF4
10	102778620	102778689	70	7	0.7111	0.0417	1.46E-23	0.6946	0.4194	2.39E-08	0.7153	0.3652	2.03E-10	-12269	on_exon	PDZD7
6	167789062	167789194	133	10	0.9265	0.2547	5.14E-68	0.7857	0.5315	4.16E-08	0.4565	0.0949	9.19E-11	-8935	on_intron	TCP10
3	55517224	55517342	119	7	0.9917	0.385	7.18E-54	0.6667	0.3203	1.36E-08	0.9756	0.6222	1.50E-09	-6748	on_intron	WNT5A
6	167530266	167530355	90	5	0.8308	0.4305	5.11E-29	0.5777	0.2524	1.00E-11	0.5673	0.2548	3.08E-12	-5059	on_intron	CCR6
10	123353267	123353349	83	5	0.8626	0.1556	2.13E-33	0.5775	0.2982	5.46E-08	0.3382	0.0457	1.00E-08	-4704	on_exon	FGFR2
7	55089734	55089778	45	5	0.9469	0.3333	3.42E-97	0.4627	0.0295	2.09E-32	0.9375	0.1293	7.32E-37	-3063	on_intron	EGFR
1	8936678	8936780	103	7	0.6619	0.0827	2.75E-31	0.8889	0.5093	2.55E-10	0.9375	0.1863	2.23E-29	-2629	on_intron	ENO1
2	176971324	176971421	98	7	0.9	0.2982	4.89E-83	0.5106	0.1622	9.27E-22	0.7186	0.2829	1.07E-20	-2476	on_intron	HOXD11
15	93017078	93017209	132	5	0.791	0.1769	2.50E-53	0.5789	0.1849	1.12E-12	0.6522	0.212	7.93E-16	-2324	on_intron	C15orf32
X	2748125	2748180	56	7	0.9095	0.0622	2.06E-86	0.7213	0.3667	2.76E-12	0.5	0.027	3.83E-26	-1350	on_exon	GYG2
3	111718457	111718564	108	7	0.9223	0.3862	5.32E-42	0.5708	0.0947	8.63E-24	0.8182	0.4155	3.23E-14	-1052	on_intron	TAGLN3
2	21266953	21267028	76	6	0.7795	0.3494	9.92E-26	0.6107	0.3121	2.61E-09	0.7119	0.4005	3.73E-12	9	upstream	APOB
6	34203665	34203774	110	11	0.8454	0.4341	3.53E-19	0.3061	0.0251	1.50E-15	0.9545	0.3074	3.87E-34	804	upstream	HMGA1
2	95960199	95960290	92	5	0.8825	0.418	1.28E-39	0.8394	0.5446	4.82E-12	0.7368	0.336	2.18E-08	2763	upstream	KCNIP3
17	38532138	38532188	51	5	0.7417	0.3155	5.73E-18	0.7196	0.2802	8.37E-19	0.4839	0.2016	2.48E-08	12072	upstream	GJD3
X	176684	176798	115	6	0.6078	0.3011	2.04E-10	0.7157	0.3421	3.67E-16	0.8407	0.4724	1.11E-09	16192	upstream	PLCXD1
7	1756030	1756166	137	8	0.6691	0.1769	1.26E-23	0.8772	0.447	9.67E-09	0.9231	0.4962	4.54E-10	22070	upstream	AC074389.6
16	10449426	10449533	108	8	0.8954	0.2199	8.40E-97	0.5198	0.2578	6.41E-12	0.4926	0.09	2.18E-32	30380	upstream	ATF7IP2

18	24796302	24796367	66	5	0.7952	0.0833	2.26E-53	0.3945	0.1193	7.70E-11	0.75	0.1	4.09E-21	31014	upstream	CHST9
4	1754314	1754400	87	5	0.634	0.3425	5.45E-11	0.7204	0.1902	4.35E-29	0.7976	0.3675	6.87E-10	31230	upstream	TMEM129
19	2364841	2364900	60	6	0.5667	0.135	4.76E-30	0.8137	0.3712	1.76E-21	0.7222	0.0955	8.95E-33	36228	upstream	LSM7
7	985719	985798	80	6	0.8411	0.3547	1.58E-29	0.5115	0.1875	2.41E-10	0.705	0.1871	4.00E-26	37038	upstream	CYP2W1
2	136836698	136836748	51	5	0.5995	0.2542	1.56E-17	0.6576	0.2885	1.22E-13	0.9677	0.6947	3.72E-11	93029	upstream	DARS
6	76875503	76875569	67	6	0.5754	0.2121	0	0.6814	0.4102	6.23E-14	0.8894	0.6052	2.57E-14	93109	upstream	IMPG1
4	187762116	187762218	103	5	0.798	0.5168	7.77E-15	0.9394	0.1468	7.23E-61	0.8491	0.1224	3.10E-14	114241	upstream	FAT1
18	77312705	77312793	89	7	0.6419	0.3877	8.49E-14	0.8073	0.4545	8.42E-20	0.6618	0.1365	1.97E-27	127009	upstream	CTDP1
20	48995552	48995661	110	7	0.5658	0.1633	0	0.5579	0.0887	6.39E-30	0.7877	0.3486	4.52E-17	131231	upstream	PTPN1
4	165729637	165729704	68	6	0.872	0.1069	1.05E-63	0.9	0.3591	5.75E-35	0.5229	0.1094	1.97E-12	148397	upstream	C4orf39
2	106959460	106959500	41	5	0.7351	0.1891	2.14E-27	0.8333	0.0728	1.71E-33	0.575	0.0952	5.29E-11	148666	upstream	UXS1
18	73323559	73323665	107	6	0.9353	0.3791	2.24E-42	0.8161	0.5149	3.74E-12	0.587	0.1667	3.57E-12	183971	upstream	C18orf62
15	93868246	93868380	135	7	0.4923	0.2067	9.50E-10	0.6875	0.1091	1.35E-17	0.427	0.0156	3.37E-10	235814	upstream	RGMA
3	76095522	76095606	85	8	0.9192	0.5958	2.83E-23	0.9674	0.5339	2.76E-16	0.8442	0.5585	5.34E-09	260789	upstream	ZNF717
1	177781960	177782056	97	6	0.3989	0.0909	5.12E-23	0.6938	0.3585	1.33E-14	0.9362	0.4854	2.14E-16	280809	upstream	RASAL2
10	119441524	119441629	106	6	0.4465	0.1019	1.91E-12	0.9624	0.6061	1.84E-15	0.3293	0.0407	2.66E-08	306547	upstream	PDZD8
8	31213098	31213165	68	6	0.75	0.3016	1.70E-23	0.6573	0.1313	2.06E-33	0.8506	0.2451	5.34E-34	321868	upstream	PURG
16	86958990	86959155	166	9	0.9245	0.0672	1.20E-43	0.4177	0.1117	2.03E-11	0.5849	0.2101	5.24E-09	358983	upstream	MTHFSD
16	86962743	86962843	101	5	0.8511	0.4167	9.33E-12	0.6809	0.1688	3.40E-16	0.6023	0.1127	7.25E-11	362736	upstream	MTHFSD
6	156717835	156717960	126	5	0.8306	0.2389	1.09E-24	0.875	0.5071	3.75E-08	0.7059	0.191	1.62E-09	381104	upstream	ARID1B
7	88361310	88361377	68	6	0.8198	0.4007	1.56E-30	0.7113	0.4009	6.89E-11	0.8333	0.5078	9.69E-11	425105	upstream	STEAP4
5	166211800	166211861	62	7	0.8646	0.0726	2.23E-74	0.6509	0.2424	1.04E-15	0.4809	0.2097	1.34E-08	499944	upstream	ODZ2
14	40470262	40470330	69	8	0.6667	0.2415	3.10E-27	0.6753	0.3057	8.01E-17	0.369	0.0672	5.72E-10	568559	upstream	FBXO33
10	123063468	123063585	118	8	0.9353	0.2222	1.16E-37	0.7	0.3901	2.89E-09	0.8765	0.1655	9.26E-27	685105	upstream	TACC2
5	30346800	30346889	90	9	0.3254	0.066	3.89E-19	0.8226	0.537	9.72E-17	0.681	0.4144	5.44E-10	846969	upstream	CDH6
10	122815914	122816048	135	5	0.8387	0.3438	2.02E-10	0.5854	0.087	2.35E-09	0.925	0.0755	6.75E-18	932642	upstream	TACC2
17	68536036	68536103	68	6	0.8718	0.4573	2.19E-20	0.822	0.5447	1.12E-11	0.8545	0.4316	1.05E-13	1212714	upstream	ABCA5
16	60357575	60357636	62	5	0.7576	0.108	3.70E-42	0.6467	0.1923	8.61E-14	0.8824	0.4565	1.03E-08	1589337	upstream	GOT2
10	58033705	58033772	68	8	0.8612	0.3394	1.67E-32	0.6286	0.3279	5.66E-15	0.8367	0.5155	6.26E-08	1995047	upstream	CISD1
