# Supplementary material for: Genome-wide methylation sequencing of paired primary and metastatic cell lines identifies common DNA methylation changes and a role for EBF3 as a candidate epigenetic driver of melanoma metastasis
Source: Oncotarget. 2016 Dec 20;8(4):6085–101. doi: 10.18632/oncotarget.14042 (PMC5351615; doi:10.18632/oncotarget.14042)
Supplement: Supplementary file 3 [file oncotarget-08-6085-s003.pdf]

Description: Excel spread sheet containing information on 10 commonly hypermethylated DMFs in all metastatic cell lines compared to its matched primary cell lines. The spreadsheet provides data of chromosome, length of the DMFs, number of CpG sites contained within the DMF, methylation value of the cell lines in the fragment (0=0% methylation, 1=100% methylation), raw P-values for each paired comparison (Fisher's exact test) , distance in relation to the gene (calculated from the start of the gene relative to the fragment. therefore negative value means the DMF is inside the gene body and the value indicates how far downstream the DMF is from the TSS, while positive value indicates the distance of the upstream DMFs to its nearest TSS), relationship of the DMFs with the gene (e.g., upstream, exon, intron) and the name of the associated gene.

#Chr	Start	End	Length of the DMFs	Number of CpG sites in the DMFs	WM115	WM266-4	P-value	Hs688(A).T	Hs688(B).T	P-value	WM75	WM373	P-value	Distance from the start of the gene	Relation with gene	Gene name
10	131763530	131763587	58	10	0.618	0.9179	2.68E-10	0.0536	0.4783	5.20E-40	0.1637	0.6538	6.15E-24	993	upstream	EBF3
11	65414680	65414818	139	14	0.5866	0.8745	5.74E-16	0.3013	0.6599	6.64E-14	0.3558	0.8153	1.25E-16	-9249	on_intron	SIPA1
17	4433208	4433295	88	5	0.2642	0.7209	3.97E-18	0.3459	0.7361	5.06E-12	0.01	0.3016	2.87E-08	-31165	on_intron	SPNS2
18	8707361	8707438	78	6	0.3113	0.9746	2.69E-44	0.0237	0.4922	1.22E-48	0.1128	0.9845	9.65E-64	-924	on_intron	CCDC165
18	8707439	8707493	55	5	0.3127	0.978	3.33E-34	0.0376	0.517	2.19E-35	0.239	0.98	3.41E-47	-979	on_intron	CCDC165
19	1030236	1030346	111	7	0.3794	0.8603	8.32E-27	0.3403	0.875	1.13E-21	0.35	0.8873	6.34E-14	-4047	on_intron	CNN2
19	14673048	14673096	49	7	0.0795	0.3545	1.06E-20	0.5093	0.7852	8.87E-12	0.0534	0.5109	1.44E-36	-32713	on_intron	TECR
20	56273715	56273790	76	6	0.0935	0.6	1.88E-41	0.0203	0.3598	6.11E-29	0.1926	0.6087	8.31E-15	-12876	on_intron	PMEPA1
5	322864	322936	73	7	0.0646	0.349	4.71E-31	0.0589	0.4316	1.57E-32	0.447	0.9615	4.95E-30	-51199	on_intron	PDCD6
6	169399692	169399797	106	6	0.1172	0.9725	2.15E-80	0.4261	0.9241	6.37E-20	0.2778	0.7745	3.10E-14	679259	upstream	DACT2
